# An integrated QTL and RNA-seq analysis revealed new petal morphology loci in *Brassica napus* L.

**DOI:** 10.1186/s13068-024-02551-z

**Published:** 2024-07-18

**Authors:** Huaixin Li, Yutian Xia, Wang Chen, Yanru Chen, Xin Cheng, Hongbo Chao, Shipeng Fan, Haibo Jia, Maoteng Li

**Affiliations:** 1 College of Life Science and Technology, Key Laboratory of Molecular Biophysics of the Ministry of Education, Huazhong University of Science and Technology, Wuhan, 430074 China; 2 School of Agricultural Sciences, Zhengzhou University, Zhengzhou, 450001 China

**Keywords:** *Brassica napus*, Petal morphology, QTL mapping, RNA-seq

## Abstract

**Background:**

Rapeseed (*Brassica napus* L.) is one of the most important oil crops and a wildly cultivated horticultural crop. The petals of *B. napus* serve to protect the reproductive organs and attract pollinators and tourists. Understanding the genetic basis of petal morphology regulation is necessary for *B. napus* breeding.

**Results:**

In the present study, the quantitative trait locus (QTL) analysis for six *B. napus* petal morphology parameters in a double haploid (DH) population was conducted across six microenvironments. A total of 243 QTLs and five QTL hotspots were observed, including 232 novel QTLs and three novel QTL hotspots. The spatiotemporal transcriptomic analysis of the diversiform petals was also conducted, which indicated that the expression of plant hormone metabolic and cytoskeletal binding protein genes was variant among diversiform petals.

**Conclusions:**

The integration of QTL and RNA-seq analysis revealed that plant hormones (including cytokinin, auxin, and gibberellin) and cytoskeleton were key regulators of the petal morphology. Subsequently, 61 high-confidence candidate genes of petal morphology regulation were identified, including *Bn.SAUR10*, *Bn.ARF18*, *Bn.KIR1*, *Bn.NGA2*, *Bn.PRF1*, and *Bn.VLN4*. The current study provided novel QTLs and candidate genes for further breeding *B. napus* varieties with diversiform petals.

**Supplementary Information:**

The online version contains supplementary material available at 10.1186/s13068-024-02551-z.

## Introduction

Rapeseed (*Brassica napus* L) is an important oil seed crop and a flower ornamented in horticulture and tourism [1, 2]. As the most attractive organ of flowers, petals serve to protect the reproductive organs (including stamens and pistils) and fascinate pollinators [3]. Hence, investigating the genetic mechanism of petal morphology formation would facilitate the breeding of *B. napus* with diversiform petals and satisfy both horticulture and agriculture demands.

As specialized leaves, the morphology of petals was determined by founder cell numbers recruited to the primordium, cell proliferation, cell expansion, and the transition from cell proliferation to cell expansion [4, 5]. The cell proliferation was determined by the cell cycle under the strict control of cyclin-dependent kinase (CDK) and cyclin (CYC) complexes, many of which have been established as organ morphology regulators [6]. For instance, CYCB1;4 was enriched in vigorously growing regions and positively regulate organ size by promoting the cell cycle in both maternal and zygotic tissues in *Arabidopsis* [7]. Other proteins (such as the *UBIQUITIN-SPECIFIC PROTEASE14* (*UBP14*)) were also demonstrated to mediate plant organ size by interacting with CDKs or CYCs [8]. The cell expansion was activated after cell proliferation, which relied on cell wall loosening and de novo synthesis of cell wall components [9, 10]. It has been proved that the overexpression *EXPANSINs* (*EXP*) could produce larger leaves with more giant cells by promoting cell wall loosening and expansion [11].

A set of petal morphogenesis regulators, spanning diverse pathways, has been discovered in *Arabidopsis*, indicating that petal morphogenesis is a complex biological process affected by multiple factors. For example, the *AUXIN RESPONSE FACTOR8* (*ARF8*) mutation in *Arabidopsis* lead to larger petals with increased cell number and cell size [12]. *TEOSINTE BRANCHED1/ CYCLOIDEA/PCF* (*TCP*) family genes are conserved regulators of plant organ growth, and the C2H2 zinc finger protein gene *RABBIT EARS* (*RBE*), as well as *miR319a*, could regulate the transcription of *TCP4* to control petal morphology [13, 14]. *JAGGED* (*JAG*) could promote petal distal growth, and narrow strap-like petals were observed in *jag* mutants [15]. It was also found that the internal-motor kinesin *AtKINESIN-13A* (*AtKIN13A*) was a negative regulator of cell expansion and cell size, and the *atkin13a* mutants had larger petals with increased cell size [16]. The Rho of Plants (ROPs) proteins could regulate the organization and dynamics of the actin and microtubule (MT) cytoskeleton, and the multiple *rop* mutants showed highly ordered cortical MT arrays and elongated petals [17]. Moreover, the accumulation of reactive oxygen species (ROS) also could moderate the shape of petal epidermis cells [18].

Petal morphology is a typical quantitative trait controlled by multiple quantitative trait loci (QTLs) [19]. QTL mapping, including linkage mapping and genome-wide association study (GWAS), was the most powerful and wildly used strategy for characterizing the quantitative traits [20, 21], which have been wildly used in garden rose [22], *Prunus mume* [23], *Arabidopsis thaliana* [24], and *B. napus* [1]. For example, major QTLs for petal number, petal size, and fragrance in the garden rose have been detected via GWAS [22]. Wang et al. investigated 20 QTLs that were significantly associated with petal size variation in two microenvironments, and identified *BnFHY3* as a negative regulator of petal size in *B. napus* among the 236 genes within the QTL confidence intervals (CIs) [3]. Qian et al. conducted GWAS for petal size in a *B. napus* natural population over three consecutive years, and 17 SNPs demonstrating significant association with petal size were identified [19]. Till now, studies focused on the petal morphology of *B. napus* were rare, and the genetic mechanism underlying remains unclear.

In this study, six petal morphology parameters were measured in a *B. napus* double haploid (DH) population consist 300 lines and subjected to QTL mapping using the high-density genetic map [25, 26]. Meanwhile, spatiotemporal transcriptomic analyses were conducted using petals with extreme morphological differences. Subsequently, candidate genes and a model of petal morphology formation were also proposed. The present results provided valuable information for breeding *B. napus* with diversiform petals.

## Materials and methods

### Plant material, genetic linkage map, and the field experiments

In this study, the KN DH (double haploid) population with the high-density genetic map was used for QTL mapping [26], and the ZS11 *B. napus* reference genome was utilized to identify genes within the QTL CIs [27]. The KN DH population was cultivated in the field of Wuhan (WH, semi-winter rape-producing areas, 113.68°E, 30.58°N) and Yangling (YL, winter rape-producing areas, 108.08°E, 34.27°N) in 2017 (17), 2018 (18), 2019 (19), and 2021 (21), respectively. Each microenvironment was abbreviated as “year & location”. For example, “17WH” represents the cultivation in Wuhan in 2017.

### Petal morphology measurement and QTL mapping

The petals of each line were collected on the day of flowering and tiled on the glass panel to capture images in real color mode at 600 dpi by the scanner. Images were analyzed by the Wseen SC-G system (Wseen, China) to collect the six petal morphology parameters, including mean petal length (MPL), mean petal width (MPW), mean petal area (MPA), mean petal perimeter (MPP), petal aspect ratio (PAR), and petal circularity degree (PCD). Among them, the PCD was calculated as follows:

$${\text{PCD}} = {4} \times {{{\text{MPA}}} \mathord{\left/ {\vphantom {{{\text{MPA}}} {\left( {\pi \times \left( {\text{major axis}} \right)^{{2}} } \right)}}} \right. \kern-0pt} {\left( {\pi \times \left( {\text{major axis}} \right)^{{2}} } \right)}}.$$

The parameters mentioned above were collected in six different microenvironments (17WH, 17YL, 18WH, 18YL, 19WH, and 19YL), which were processed to QTL mapping subsequently using Windows QTL Cartographer 2.5 software under the composite interval mapping (CIM) method [28]. Significance levels for the LOD scores were determined by a 1000-permutation test based on a 5% experiment-wise error rate, and an LOD score threshold of 2.5 was used to identify significant QTLs. The QTL integration was processed by BioMercator V4.2 program via meta-analysis [29]. Significant QTLs detected by Windows QTL Cartographer 2.5 were called identified QTLs, which were named with the prefix “q”, followed by the trait name, microenvironment abbreviation, and chromosome number. Furthermore, integrated QTLs that control the same trait under different microenvironments were called consensus QTLs and named with the prefix “cq”, followed by the trait name and chromosome number. Identified QTLs that cannot integrate with others were also taken as consensus QTLs to facilitate description. Consensus QTLs with overlapped CIs were integrated into unique QTLs and named with the prefix “uq”, followed by the chromosome number. A serial number will be suffixed if more than one QTL is detected in the same chromosome. For example, *qMPA-17WH6-1* represents the first identified QTL on chromosome A06 that controlled MPA in the 17WH microenvironment.

In the present study, QTLs with over 15% of the PV (phenotypic variation) under one microenvironment or with over 10% of the PV that could be detected in more than one microenvironment were called major QTLs.

### RNA-seq samples and differential gene expression analysis

The petals of two days before flowering (bud stage, abbreviated to “B”) and the day of flowering (flower stage, abbreviated to “F”) of I-065 (big round petal, BP), I-120 (small round petal, SP), and I-059 (elongated petal, LP) were collected in 21WH, which were cut into three separated parts including the distal, the middle, and the proximal, respectively (Additional file 1**: Fig. S10**). A total of 18 samples were collected with three repeats, frozen in liquid nitrogen immediately, and stored in the -80℃ refrigerator for RNA-seq analysis. The RNA-seq samples were named after “petal-type & development stage & part & replications”. In addition, “distal”, “middle”, and “proximal” were abbreviated as “D”, “M”, and “P”, respectively. For example, BP^F−D^-1 represents the first replication of the distal of the big petal collected on the day of flowering.

The total RNA extraction, reverse transcription, and RNA-seq were conducted following the reported process [30]. The raw data of RNA-seq have been uploaded to the NCBI Sequence Read Archive (SRA, https://www.ncbi.nlm.nih.gov/sra) with the accession number PRJNA1052045. The gene expression level was evaluated by fragments per kilobase of exon model per million mapped fragments (FPKM), and the differential expressed genes (DEGs) were identified by calculating the log2 (fold change) of FPKMs between two samples. The thresholds for DEG identification were set at |log2(fold change)|> 1, p-value < 0.05, and q-value < 0.05. Moreover, the comparison of gene expression between samples was denoted as “A vs B”, indicating the expression level of sample B relative to sample A.

### The evaluation of RNA-seq via quantitative real-time PCR (qRT-PCR)

Total RNA was extracted and reverse transcripted, as mentioned before. Seven genes were randomly selected for qRT-PCR analysis, together with the internal reference gene *TIP41* (*TAP42 INTERACTING PROTEIN OF 41 KDA*), using the qPCR SYBR Green Master Mix (Yeasen, China) and QuantStudio3 Real-Time PCR System (ABI). Primers for qRT-PCR were designed using Oligo7 software, and all primers used in qRT-PCR are listed in Additional file 2**: Table S13**.

### The epidermal cell morphology analysis using the scanning tunneling microscope (STM)

The petals on the bud and flower stage of BP, SP, and LP were collected in 21WH, which were immersed into Karnovsky fixative solution and stored in the 4℃ refrigerator. Petals mentioned above were gradient dehydration under 10% ~ 100% tertiary butanol solutions and were subsequently vacuum freeze-dried overnight for STM analysis. The images of epidermal cells were analyzed using ImageJ software. The morphology of epidermal cells from six different areas of the petals was measured, including the center of the petal distal (DC), the edge of the petal distal (DE), the center of the petal middle (MC), the edge of the petal middle (ME), the center of the petal proximal (PC), and the edge of the petal proximal (PE), respectively. The epidermal cell number of each petal part was calculated by dividing the area of this part by the corresponding average cell area. The naming scheme of STM samples was “petal-type & development stage & area”. For example, “BP^F−DC^” represents the center of the petal distal of the big petal collected on the day of flowering.

## Results

### The petal morphology of *B. napus* is a stable inherited quantitative trait

**Fig. 1:**
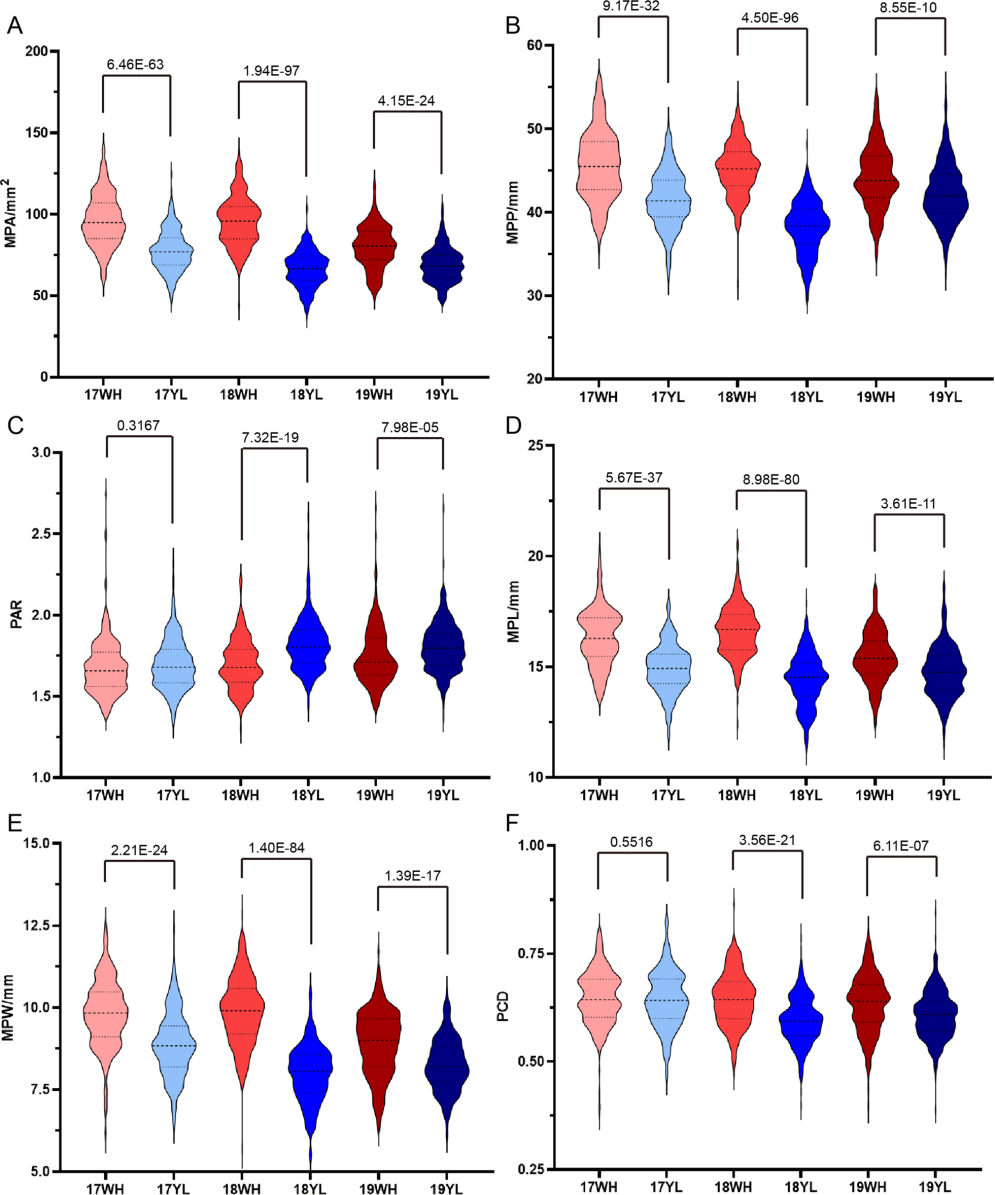
The distribution of six petal morphology parameters. The phenotype distribution of MPA (**A**), MPP (**B**), PAR (**C**), MPL (**D**), MPW (**E**), and PCD (**F**) in the KN DH population under six microenvironments including 17WH, 17YL, 18WH, 18YL, 19WH, and 19YL. The two-tailed one-way ANOVA was used to evaluate the variance between WH and YL in the same year, and the p-value was annotated above the violin plot

The phenotypes of the six petal morphology parameters of the KN DH population and their parental lines were collected under six microenvironments, and a wide range of variations were observed. For example, the maximum of MPA in 19WH was 119.29 mm^2^, which was 2.41 times of the minimum (49.50 mm^2^) (Additional file 2: Table S1). Further analysis revealed that all six parameters exhibited transgressive segregation and continuous frequency distribution, manifesting as quantitative traits that suit QTL mapping analysis (Fig. 1; Additional file 1: Fig. S1-3; Additional file 2: Table S1). The correlation analysis showed that all of the six parameters were significantly correlated. For example, MPA was significantly positively correlated with MPP, MPL, MPW, and PCD in 17WH with the Pearson correlation coefficients of 0.904, 0.783, 0.921, and 0.424, respectively. In contrast, MPA was significantly negatively correlated with PAR in 17WH with the Pearson correlation coefficients of -0.404 (Additional file 2: Table S2-7).

In addition, apparent distribution divergences of the six parameters were observed between winter and semi-winter environments. Specifically, the MPA, MPL, MPW, PAR, and PCD of WH were larger than that of YL in the same year, while the MPP was just the opposite way. All divergences mentioned above reached a significant level (Fig. 1), indicating that larger and rounder petals of *B. napus* were usually generated in the semi-winter environment compared to the winter environment. The broad-sense heritability of MPA, MPP, PAR, MPW, MPL, and PCD was 74.35%, 74.38%, 87.49%, 80.78%, 78.06%, and 83.64%, respectively (Additional file 2: Table S1), which manifested that though significantly affected by the environments, the petal morphology of *B. napus* could be stably inherited.

### Identification and integration of QTLs for petal morphology in *B. napus*.

A total of 43, 42, 29, 48, 43, and 38 identified QTLs were detected for MPA, MPP, MPL, MPW, PAR, and PCD, respectively, which were distributed on all of the 19 chromosomes except for ChrA02 and ChrA05 (Additional file 2: Table S8, Additional file 1: Fig. S4). From the microenvironment point of view, 18YL consists of the highest number of identified QTLs (51), followed by 18WH (45), 17WH (44), 17YL (39), 19YL (34), and 19WH (30). Among them, 126 and 117 identified QTLs were distributed on the A and C subgenomes, respectively. In addition, ChrA01 contained the highest number of identified QTLs (48), followed by ChrA06 (45) and ChrA09 (44), indicating that these three chromosomes played a vital role in the formation of petal morphology in *B. napus*.

The identified QTLs controlling the same trait in different microenvironments were integrated, and 211 consensus QTLs were detected, including nine major QTLs and 28 environmentally stable QTLs that could be consistently detected in more than one microenvironment. Among the 28 environmental stable consensus QTLs, seven QTLs (*cqMPA-6–6*, *cqMPA-6–7*, *cqMPA-6–8*, *cqMPA-6–9*, *cqMPA-6–10*, *cqMPA-9–2*, and c*qMPA-11–11*) controlled MPA, three QTLs (*cqMPP-6–6*, *cqMPP-9–1*, and *cqMPP-11–5*) controlled MPP, six QTLs (*cqMPL-9–2*, *cqMPL-9–3*, *cqMPL-11–1*, *cqMPL-11–3*, *cqMPL-16–3*, and *cqMPL-17–2*) controlled MPL, two QTLs (*cqMPW-6–3* and *cqMPW-9–1*) controlled MPW, six QTLs (*cqPAR-3–2*, *cqPAR-3–9*, *cqPAR-3–10*, *cqPAR-9–1*, *cqPAR-9–2*, and *cqPAR-9–4*) controlled PAR, and four QTLs (*cqPCD-6–1*, *cqPCD-9–3*, *cqPCD-9–9*, and *cqPCD-18–1*) controlled PCD (Additional file 2: Table S9).

**Fig. 2:**
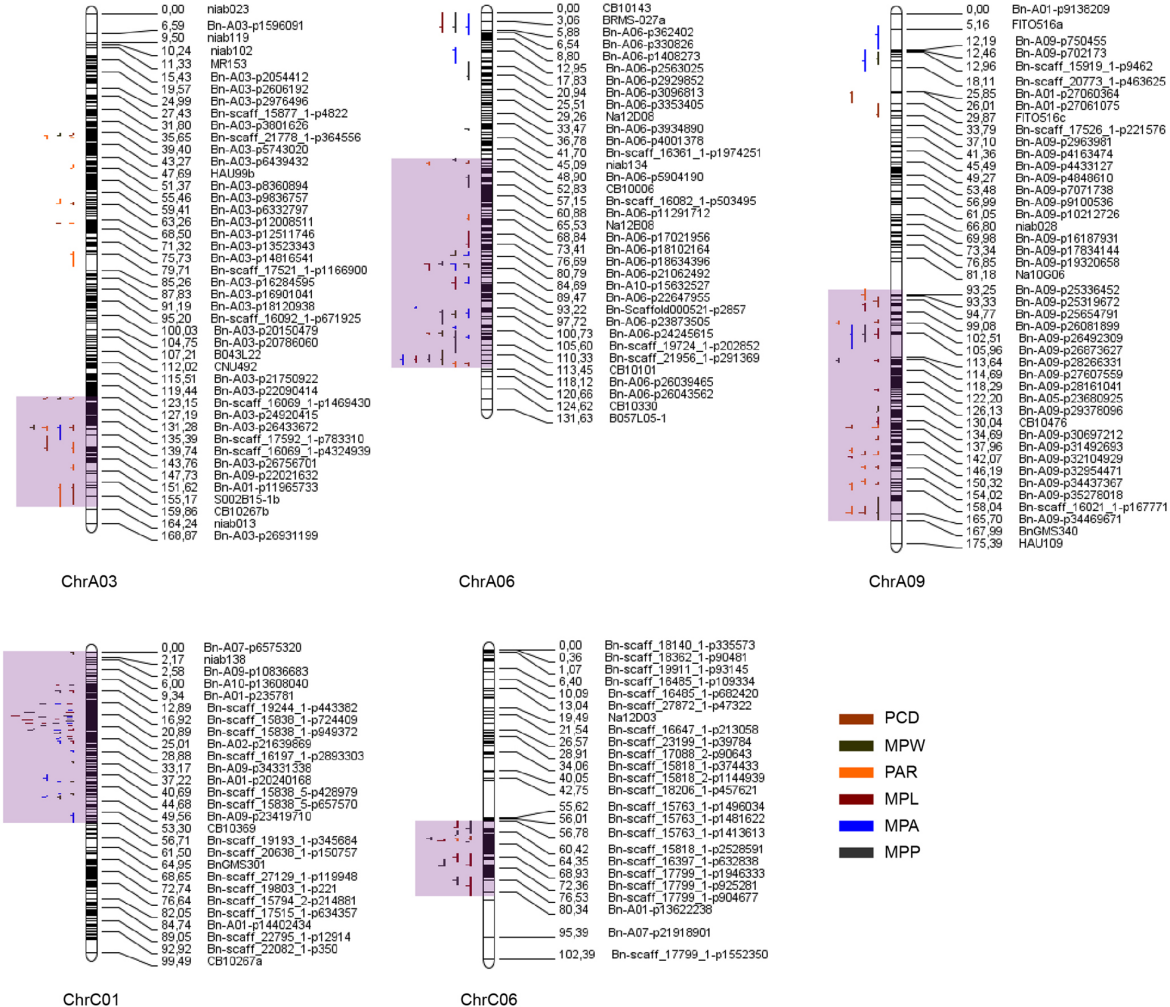
The QTL distribution on ChrA03, ChrA06, ChrA09, ChrC01, and ChrC06. The identified QTLs of MPA, MPP, PAR, MPL, MPW, and PCD in the ChrA03, ChrA06, ChrA09, ChrC01, and ChrC06 QTL hotspots. And the QTL hotspots were highlighted in the purple boxes

Since the six parameters were significantly correlated, consensus QTLs were further integrated into unique QTLs to discover the QTL hotspots for petal morphology. It turned out that 138 consensus QTLs with overlapping CIs could be integrated into 58 unique QTLs, each controlling more than one petal morphology parameter (Additional file 2: Table S10). Moreover, five QTL hotspots located on ChrA03, ChrA06, ChrA09, ChrC01, and ChrC06 were observed (Fig. 2). DH lines that inherited the genic background from Ken-C8 and N53-2 (named DH^Ken−C8^ and DH^N53−2^, respectively) in the five QTL hotspots were isolated, and their PCD were compared, respectively. For the ChrA03 and ChrA06 QTL hotspots, the PCD of DH^Ken−C8^ was significantly higher than that of DH^N53−2^ in all six microenvironments. For the ChrA09 QTL hotspots, the PCD of DH^Ken−C8^ was significantly lower than that of DH^N53−2^ in all six microenvironments. For the ChrC01 QTL hotspot, the PCD of DH^Ken−C8^ was significantly higher than that of DH^N53−2^ in three of the six microenvironments. As for the ChrC06 QTL hotspot, the PCD of DH^Ken−C8^ was significantly lower than that of DH^N53−2^ in four out of the six microenvironments (Additional file 1: Fig. S5), which indicated a strong and stable effect of the five QTL hotspots on petal morphogenesis.

### Candidate gene identification within QTLs

According to the collinearity of the high-density genetic map and ZS11 *B. napus* reference genome, 25,437 annotated genes were located in the QTL CIs, among which 5292 were located in the QTL hotspots on ChrA03, ChrA06, ChrA09, and ChrC01. KEGG and GO enrichment analysis for the 25,437 genes revealed that the top three significantly enriched GO terms were “nuclear transcription factor complex”, “RNA polymerase II transcription factor complex”, and “transcription factor complex”, and the top three significantly enriched KEGG terms were “Sesquiterpenoid and triterpenoid biosynthesis”, “Lysosome”, and “Sulfur metabolism”, respectively (Additional file 1: Fig. S7). Meanwhile, enrichment analysis for the 5292 genes within the QTL hotspots showed that “Plant hormone signal transduction” was the most significant KEGG term, containing the highest number of genes, and “protein phosphatase type 2A complex” was one of the significantly enriched GO terms. Thus, the plant hormones and MT were of great importance for petal morphogenesis (Additional file 1: Fig. S6).

At the same time, it was noticed that “nuclear DNA-directed RNA polymerase complex”, “RNA polymerase complex”, “DNA-directed RNA polymerase complex”, “RNA polymerase II, holoenzyme”, and “transferase complex, transferring phosphorus-containing groups” were the most significantly enriched GO terms of the 5292 genes (Additional file 1: Fig. S6A), which indicated that complex transcriptional regulations were involved in *B. napus* petal morphogenesis. Thus, the transcriptional factors within the QTL CIs were screened, and a total of 1846 TFs belonging to 55 TF families were identified, among which 412 TFs belonging to 48 TF families were in the QTL hotspots (Additional file 1: Fig. S17A). Meanwhile, 379 *Arabidopsis* genes that have been reported as plant organ development regulators (PDRs) were collected, which were homology with 1410 *B. napus* genes (Additional file 2: Table S11).

**Fig. 3:**
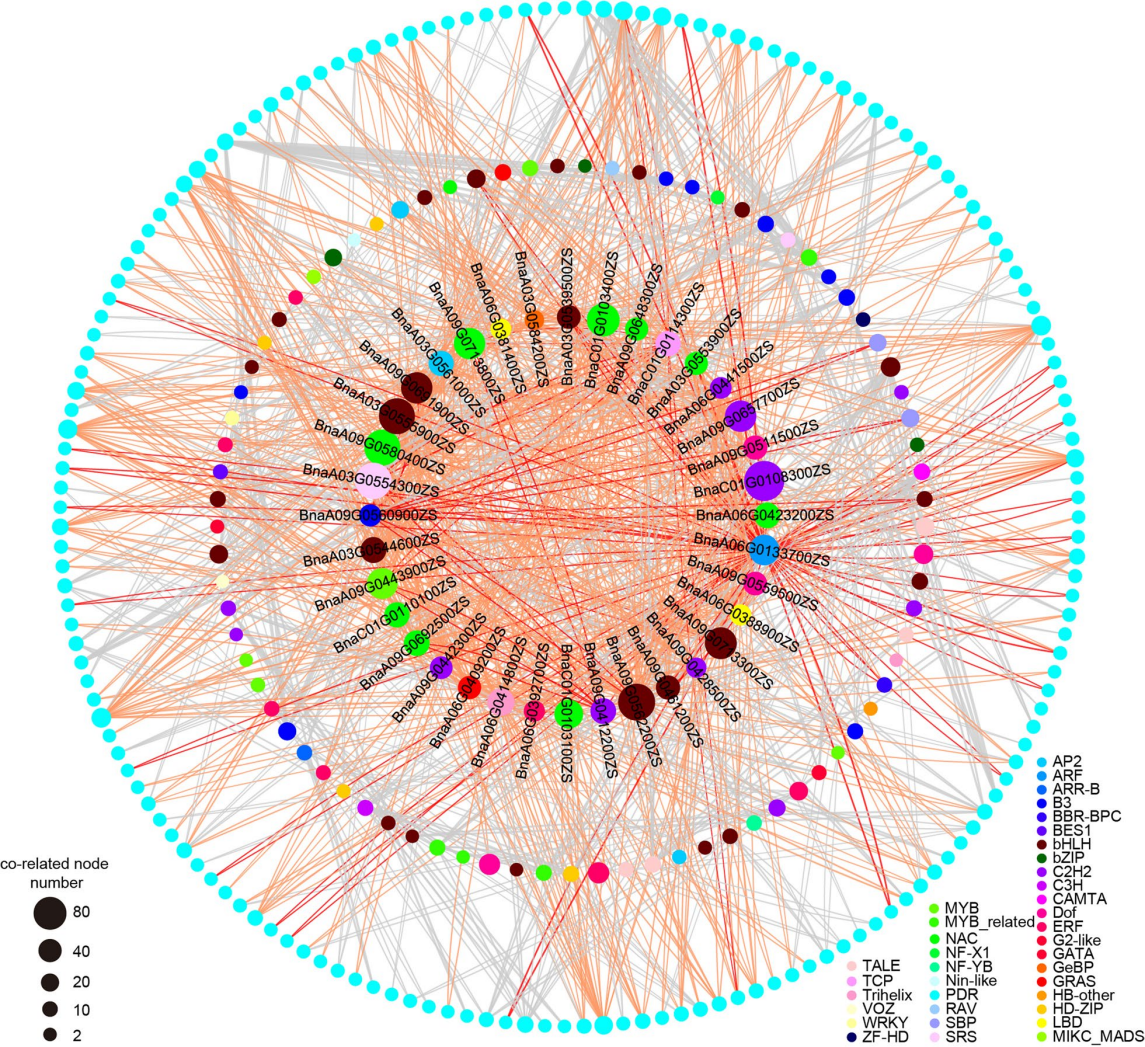
The network of 117 TFs in QTL hotspots and PDRs. The color of the node represents different TF families, and the size of node represent the number of co-related nodes. The orange line represents the network of 36 TFs that are co-related with more than 20 PDRs (except the four PDR TFs: *BnaA06G0133700ZS* (*ARF22*), *BnaA09G0412200ZS* (*TCP24*), *BnaC01G0114300ZS* (*TCP2*), and *BnaA09G0560900ZS* (*NGATHA2*)), and the red line represents the network of *ARF22*, *TCP24*, *TCP2*, and *NGATHA2*

The network of the 412 TFs distributed in the QTL hotspots and these PDRs was screened using the String database (https://cn.string-db.org/), and there were 117 TFs belonging to 36 TF families co-related with PDRs (Fig. 3). Among them, the bHLH TF family comprised the largest number of TFs (24), followed by C2H2 (11) and NAC (10) (Additional file 1: Fig. S17A). There were 36 TFs co-related with more than 20 PDRs, and 33 of them contained sequence variations between Ken-C8 and N53-2 within genes or the 2 kb flanks (Additional file 2: Table S15), which were identified as candidate TF genes of petal morphology regulation in *B. napus*. Moreover, four out of the 33 candidate TFs were PDRs, including *BnaA06G0133700ZS* (*ARF22*), *BnaA09G0412200ZS* (*TCP24*), *BnaC01G0114300ZS* (*TCP2*), and *BnaA09G0560900ZS* (*NGATHA2*), which verified the reliability of the analysis. In addition, 353 PDRs were distributed in the QTLs, and 289 of them contained sequence variations between Ken-C8 and N53-2 within genes or the 2-kb flanks, which were also identified as candidate genes of petal morphology regulation in *B. napus*.

### STM analysis revealed tremendous epidermal cell morphology variance of different variform *B. napus* petal

**Fig. 4:**
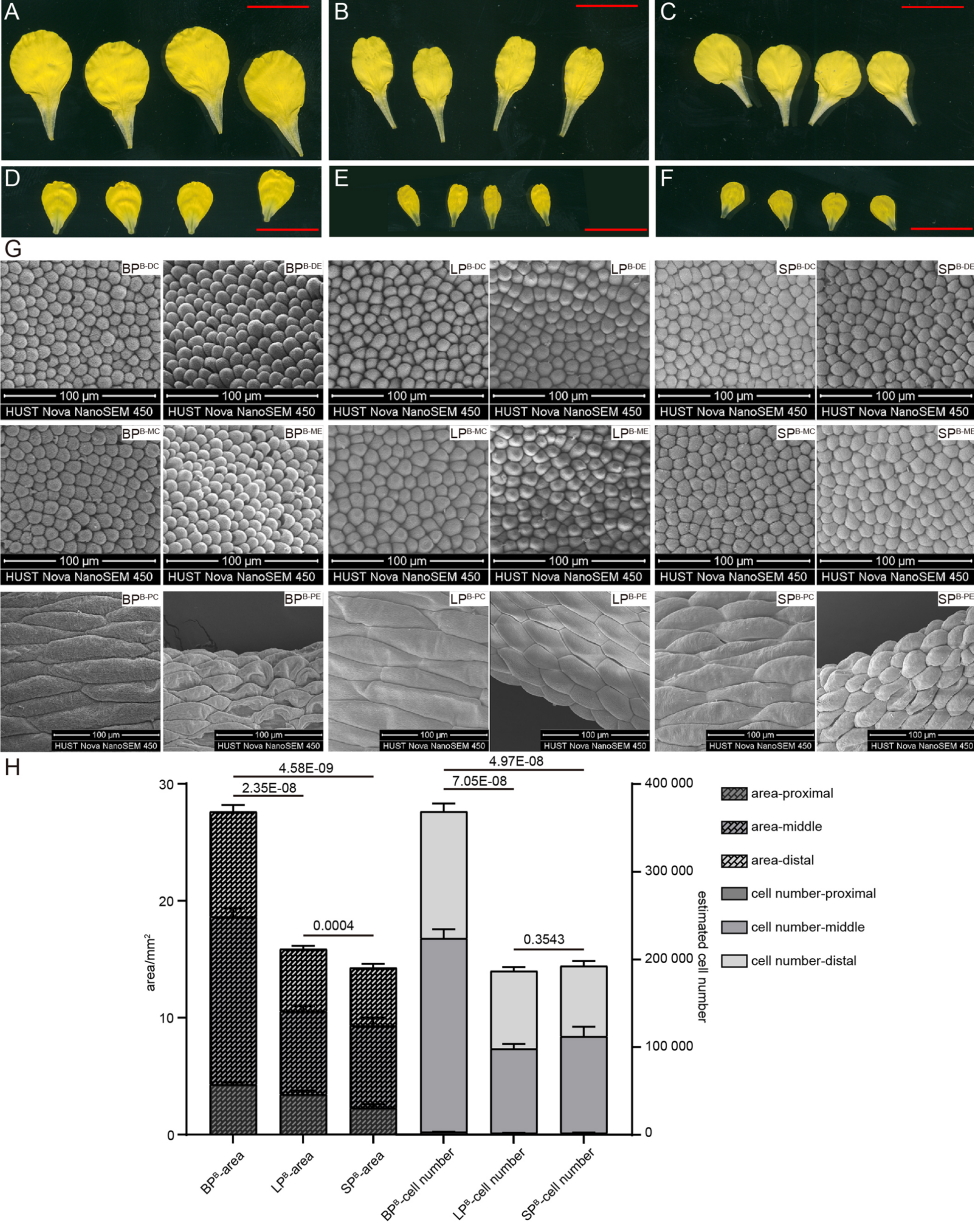
The pictures and STM images of diversiform petals. The picture of BP^F^ (**A**), LP^F^ (**B**), SP^F^ (**C**), BP^B^ (**D**), LP^B^ (**E**), and SP^B^ (**F**), and the red bars represent 10mm. **G** The STM images of the epidermis cell of different petals and parts in the bud stage (annotated in the upper right corner). **H** The area (left y-axis) and estimated epidermis cell number (right y-axis) of different petals and parts in the bud stage. The significance of area and cell number variations of the entire petal between diverse forms of petals were verified by two-tailed one-way ANOVA, respectively, and the p-values were annotated above the histograms

The organ morphology was directly determined by the epidermal cell number and morphology. Therefore, to characterize the petal morphology variation of the *B. napus*, three lines from the KN DH population, each exhibiting distinct petal morphologies (including BP, LP, and SP), were selected for STM analysis. The morphology of epidermal cells was measured in six different areas. It was revealed that dramatic epidermal cell size variations were observed on the different areas of the petals. Specifically, for the BP^F^, the cell size of BP^F−MC^ was larger than that of BP^F−DC^ by 7.24%, and the cell size of BP^F−ME^ was larger than that of BP^F−DE^ by 2.94%. In addition, the cell size of BP^F−ME^ was larger than that of BP^F−MC^ by 13.45%, and the cell size of BP^F−DE^ was larger than that of BP^F−DC^ by 18.18%. While the epidermal cell size of BP^F−PC^ was 40.08-fold of that of BP^F−MC^, and the epidermal cell size of BP^F−PE^ was 15.95-fold of that of BP^F−ME^, at the same time, the epidermal cell size of BP^F−PC^ was 121.52% larger than that of BP^F−PE^. The same phenomenon was observed on the LP^F^, SP^F^, BP^B^, LP^B^, and SP^B^ (Fig. 4; Additional file 1: Fig. S8, 9).

The epidermal cell size variations between variform petals were also investigated. Though the area of BP^F^ was 1.65 and 1.91 times that of LP^F^ and SP^F^, the epidermal cell size difference among them was minimal, especially on the distal and middle parts. The same phenomenon was observed on BP^B^, LP^B^, and SP^B^ (Fig. 4; Additional file 1: Fig. S8, 9). In detail, the cell size variation between BP^F^ and SP^F^ on the DC, DE, MC, and ME were 4.18%, 15.90%, 8.55%, and 22.61%, respectively. The cell size variation between BP^B^ and SP^B^ on the DC, DE, MC, and ME were -0.06%, 1.09%, 10.06%, and -6.13%, respectively. While the epidermal cell size of BP^F−PC^ was 1.69 and 1.67 times that of LP^F^ and SP^F^, and the epidermal cell size of SP^F−PE^ was only about half of that of BP^F^ and LP^F^, which indicated that the MPA variations among variform petals mainly result from the difference in cell number rather than cell size, especially on the distal and middle parts. Therefore, the estimated cell numbers of the distal, middle, proximal, and the entire petal of BP, LP, and SP were further calculated and compared, which revealed that the cell number of BP^B^ was 1.97 and 1.91 times that of LP^B^ and SP^B^, respectively, and the cell number of BP^F^ was 1.83 and 1.76 times that of LP^F^ and SP^F^, respectively (Fig. 4H; Additional file 1: Fig. S8B). In summary, it was estimated that the cell number accounts for 92.15% of the petal size variation between BP^F^ and SP^F^, while cell size accounts for the remaining 7.85%.

The epidermal cell shape was also investigated. Generally, all epidermal cells were round or near-round, except for those in the proximal parts, which were fusiform. Consistent with the slender shape of LP, the epidermal cell length of LP^F−MC^ was larger than that of BP^F−MC^ and SP^F−MC^ by 27.81% and 26.25%, respectively (Additional file 1: Fig. S9E). Thus, cell polar expansion should contribute to the slender morphology of petals.

The cell epidermal width of the middle parts was also measured, which defined the MPW together with the cell number on the horizontal axis. It was revealed that though the width of differently variform petals was varying (BP > SP > LP), their epidermal cell width in the middle part was almost consistent. The epidermal cell width of LP^F−ME^ was slightly larger than that of BP^F−ME^ and SP^F−ME^ (Additional file 1: Fig. S8C), which indicate that it was the cell number rather than the cell width variation on the horizontal axis that caused the narrow width of LP.

### Spatiotemporal transcriptomic analysis revealed GAs, as well as IAA and zeatin, are essential for petal morphogenesis

**Fig. 5:**
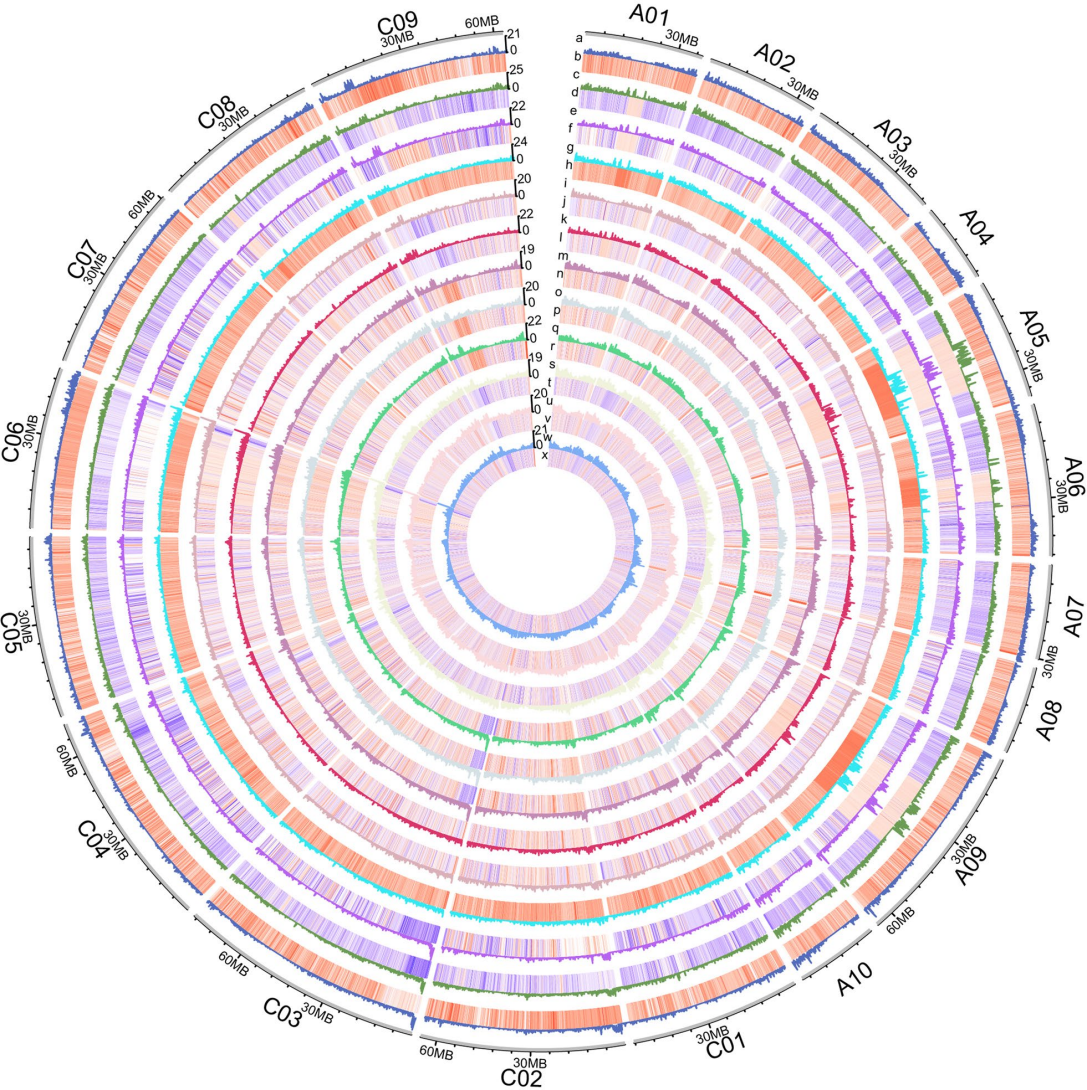
The overview of spatiotemporal transcriptomic analysis of the different parts of diversiform petals. The meaning of each circle was as follows: a: the DEGs density of BP^B−D^ vs LP^B−D^; b: the heat map of DEGs of BP^B−D^ vs LP^B−D^; c: the DEGs density of BP^B−M^ vs LP^B−M^; d: the heat map of DEGs of BP^B−M^ vs LP^B−M^; e: the DEGs density of BP^B−P^ vs LP^B−P^; f: the heat map of DEGs of BP^B−P^ vs LP^B−P^; g: the DEGs density of BP^B−D^ vs SP^B−D^; h: the heat map of DEGs of BP^B−D^ vs SP^B−D^; i: the DEGs density of BP^B−M^ vs SP^B−M^; j: the heat map of DEGs of BP^B−M^ vs SP^B−M^; k: the DEGs density of BP^B−P^ vs SP^B−P^; l: the heat map of DEGs of BP^B−P^ vs SP^B−P^; m: the DEGs density of BP^F−D^ vs LP^F−D^; n: the heat map of DEGs of BP^F−D^ vs LP^F−D^; o: the DEGs density of BP^F−M^ vs LP^F−M^; p: the heat map of DEGs of BP^F−M^ vs LP^F−M^; q: the DEGs density of BP^F−P^ vs LP^F−P^; r: the heat map of DEGs of BP^F−P^ vs LP^F−P^; s: the DEGs density of BP^F−D^ vs SP^F−D^; t: the heat map of DEGs of BP^F−D^ vs SP^F−D^; u: the DEGs density of BP^F−M^ vs SP^F−M^; v: the heat map of DEGs of BP^F−M^ vs SP^F−M^; w: the DEGs density of BP^F−P^ vs SP^F−P^; x: and the heat map of DEGs of BP^F−P^ vs SP^F−P^. Among them, the heat maps were drawn using log2(fold change) of FPKM, and the DEG densities were calculated with a sliding window of 100 Kbps and a step size of 10 Kbps

**Fig. 6:**
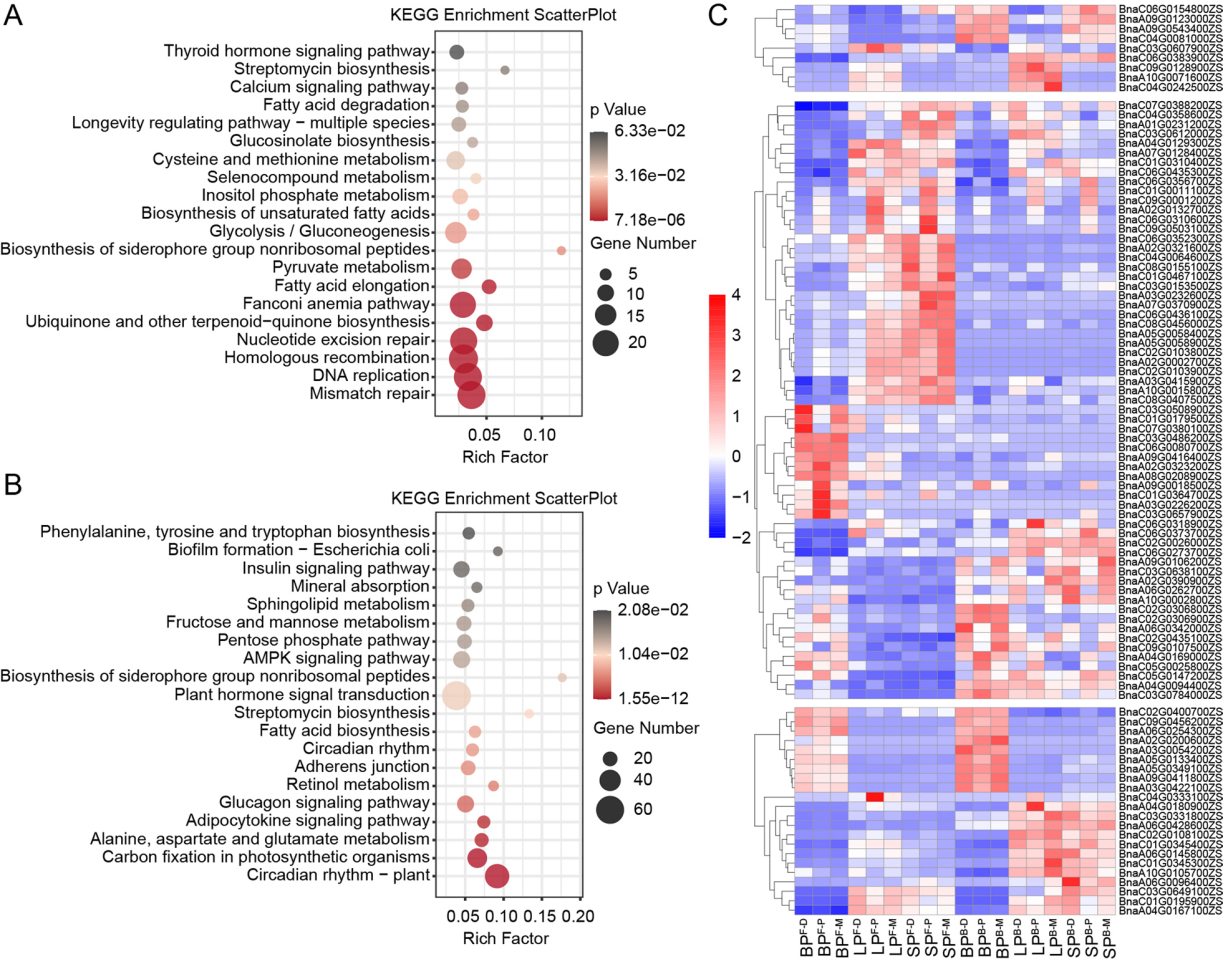
The expression and enrichment analysis of common DEGs of diversiform petals.** A** The KEGG enrichment analysis of common DEGs of BP^B−D^ vs LP^B−D^, BP^B−D^ vs SP^B−D^, BP^B−M^ vs LP^B−M^, BP^B−M^ vs SP^B−M^, BP^B−P^ vs LP^B−P^, and BP^B−P^ vs SP^B−P^. **B** The KEGG enrichment analysis of common DEGs of BP^F−D^ vs LP^F−D^, BP^F−D^ vs SP^F−D^, BP^F−M^ vs LP^F−M^, BP^F−M^ vs SP^F−M^, BP^F−P^ vs LP^F−P^, and BP^F−P^ vs SP^F−P^. **C** The heat map of genes enriched in the terms of “cytoskeletal protein binding” (the upper section), “Plant hormone signal transduction” (the middle section), and “DNA replication” (the lower section) in Figure A and Figure B, which was drawn using their FPKMs that normalized by z-score

To identify DEGs associated with petal morphology, RNA-seq analysis was conducted on the distal, middle, and proximal parts of petals with different morphologies (including BP, LP, and SP) and developmental stages (including the bud and flower stage) (Fig. 5; Additional file 1: Fig. S10, 11). Overall, 42.14 to 48.11 M raw reads and 41.69 to 47.62 M clean reads were generated for each sample, with the Q20 value larger than 94% and the Q30 value larger than 85%, respectively. In addition, the total mapping radio of each sample ranged from 85.68% to 89.25% (Additional file 2: Table S12). Seven genes were randomly selected for qRT-PCR analysis, and it was revealed that their expression tendency is consistent with the RNA-seq result (Additional file 1: Fig. S12). The abovementioned parameters indicated that the RNA-seq data obtained in the present study were of high quality and accurate, suitable for further analysis. Since STM analysis indicated that the cell number of BP was larger than that of LP and SP, which is the main factor of petal morphology variations in *B. napus*, common DEGs of BP^B−D^ vs LP^B−D^, BP^B−D^ vs SP^B−D^, BP^B−M^ vs LP^B−M^, BP^B−M^ vs SP^B−M^, BP^B−P^ vs LP^B−P^, and BP^B−P^ vs SP^B−P^ were screened out. A total of 1016 common DEGs (including 444 down-regulated DEGs and 572 up-regulated DEGs) were observed (Additional file 1: Fig. S13A, B), and the top four significantly enriched KEGG terms of them were “Mismatch repair”, “DNA replication”, “Homologous recombination”, and “Nucleotide excision repair” (Fig. 6A, C), suggesting higher DNA replication activity in BP^B^ compared to LP^B^ and SP^B^. “Pyruvate metabolism”, “Biosynthesis of unsaturated fatty acids”, “Inositol phosphate metabolism”, and “Cysteine and methionine metabolism” were also significantly enriched (Fig. 6A), which indicated distinct metabolic profiles among diverse petal types in the bud stage. In addition, the common DEGs of BP^F−D^ vs LP^F−D^, BP^F−D^ vs SP^F−D^, BP^F−M^ vs LP^F−M^, BP^F−M^ vs SP^F−M^, BP^F−P^ vs LP^F−P^, and BP^F−P^ vs SP^F−P^ were also investigated. A total of 2205 common DEGs (including 1066 down-regulated DEGs and 1139 up-regulated DEGs) were observed (Additional file 1: Fig. S13C, D). And the KEGG analysis of them revealed that “Plant hormone signal transduction” was significantly enriched and comprised the highest number of DEGs (63), among which 23 were annotated related with IAA signal transduction (Fig. 6B, C). Additionally, “Fatty acid biosynthesis”, “Alanine, aspartate and glutamate metabolism”, “Fructose and mannose metabolism”, “Starch and sucrose metabolism”, “Tryptophan metabolism”, and “Sphingolipid metabolism” were also significantly enriched, which was quite different from the significantly enriched KEGG terms in the bud stage petals and indicated substantial metabolic changes during the development of diversiform petals (Fig. 6B).

Since both BP and SP were rounded petals, whereas LP was slender, to dissect the mechanism of such petal shape variations, DEGs that consistently detected between BP and LP on the three parts and two stages were isolated for enrichment analysis, while the common DEGs consistently detected between BP and SP on the three parts and two stages were excluded meanwhile. A total of 1743 DEGs (including 1009 down-regulated DEGs and 734 up-regulated DEGs) were screened out (Additional file 1: Fig. S14A), and the most significantly enriched GO terms were “actin binding” and “cytoskeletal protein binding”, which consist of nine genes. The nine genes were differently expressed in LP compared to BP and SP across both bud and flower stages, indicating that the cytoskeleton plays a crucial role in LP formation (Fig. 6C; Additional file 1: Fig. S14B).

The expression profile of genes involved in the DNA replication was further investigated, which revealed higher expression of genes encoding DNA polymerase α-primase complex, DNA polymerase δ complex, and DNA polymerase ε complex genes in BP^B^ compared to LP^B^ and SP^B^, especially on the distal and middle parts (Additional file 1: Fig. S15). Moreover, the expression of genes involved in the plant hormone synthesis showed that the two most critical GA-activating enzyme genes, *GA20ox* and *GA3ox* [31], were highly expressed in the BP^B^ compared to both LP^B^ and SP^B^ (Additional file 1: Fig. S16A, B), which indicated that the GAs contribute to the cell number and cell size variation between BP, LP, and SP. Previous study pointed out that CKXs (CYTOKININ OXIDASEs) could directly affect the zeatin concentration in plants [32]. In the present study, the expression of *CKXs* genes in BP^B^ was consistently lower than that of SP^B^ and LP^B^, with the highest expression of *CKXs* observed in LP^B−D^ and LP^B−M^ (Additional file 1: Fig. S16C). This pattern aligned with the observed variations in cell number among petals of different morphologies, indicating that the zeatin could regulate the petal morphology by regulating cell number in the bud stage.

The expression profile of genes that participated in the plant hormone signal transduction revealed that the most pronounced and stable expression variation was observed in the auxin signal transduction (Additional file 1: Fig. S16E). Moreover, the expression of auxin signal transduction genes in BP^F^ was consistently higher than that of LP^F^ and SP^F^ across all three parts of the petal. The expression of *YUCCAs*, the rate-limiting enzyme gene of IAA synthesis [33], was higher in BP^F^ and LP^F^ compared to SP^F^, with the highest expression observed in the BP^F−P^ (Additional file 1: Fig. S16D). This may explain why the cell size of BP^F−PC^ was much larger than others and why the cell size of BP^F^ and LP^F^ was larger than that of SP^F^. Thus, these findings suggest that IAA was also involved in the petal morphogenesis.

### Novel regulation model and candidate genes for petal morphology in *B. napus* through the integration of QTL mapping and RNA-seq

Among the 289 candidate PDR genes within QTL CIs, 152 were expressed in the flower stage petals (FPKM ≥ 1), including 65 PDRs that exhibited consistent expression variation between BP^F^ and the other two types of petals (Additional file 2: Table S16). Moreover, 168 out of the 289 PDRs were expressed in the bud stage petals (FPKM ≥ 1), including 56 PDRs that exhibited consistent expression variation between BP^B^ and the other two types of petals (Additional file 2: Table S17). Additionally, 21 out of the 289 PDRs showed consistent expression variation between BP and the other two types of petals in both the bud and flower stages, which were taken as high-confidence candidate genes of petal morphology formation (Additional file 2: Table S14). The expression of 33 candidate TFs was also investigated, among which 19 were expressed in the bud or flower stage petals of *B. napus* (Additional file 1: Fig. S17B), and those 19 TFs were also considered high-confidence candidate genes of petal morphology formation, including four PDRs and 15 novel genes (Additional file 2: Table S14).

Besides, since QTL mapping and RNA-seq indicated that plant hormone signal transduction was the most critical pathway in petal morphology regulation, 564 genes participated in the signal transduction of IAA, CK, or GAs signals were identified based on their KEGG annotation. Among them, 123 genes were in the QTL CIs and contained sequence variations between Ken-C8 and N53-2 within genes or the 2 kb flanks. RNA-seq revealed that seven out of the 123 genes performed consistent expression variation between BP and the other two types of petals in both the bud and flower stages. These seven genes were also considered high-confidence candidate genes of petal morphology formation (Additional file 2: Table S14). As expected, one of the seven genes (*ARF18*, *BnaA09G0559300ZS*) was among the PDRs described above, which proved the effectiveness of the high-confidence candidate gene selection and further confirmed the role of auxin in petal morphogenesis. Additionally, 11 expressed genes in petals encode key enzymes of IAA, zeatin, or GAs metabolism were located in QTL CIs (including five *YUCCA* genes, three *CKX* genes, two *GA2ox* genes, and one *GA20ox* gene), which were also considered high-confidence candidate genes (Additional file 2: Table S14). Moreover, considering the non-negligible effect of the cytoskeleton on slender petal shape formation, 18 cytoskeleton genes located in the QTL CIs were identified, three of which overlapped with the nine “actin binding” and “cytoskeletal protein binding” genes described above, which were also taken as high-confidence candidate genes. In total, 61 high-confidence candidate genes were identified.

Among the 61 high-confidence candidate genes, *BnaC03G0508900ZS* homology with the SMALL AUXIN UPREGULATED RNA 10 (SAUR10) gene *AT2G18010*. In previous studies, SAUR10 was induced by auxin and brassinosteroids, which respond to light conditions and affect the branch angle [34], while it has not been reported to affect organ size. In the present study, *BnaC03G0508900ZS* was located in the ChrA03 QTL hotspot, with its expression in BP consistently higher than in LP and SP. In addition, 14 SNPs and 5 Indels were detected within *BnaC03G0508900ZS* and its 2-kb flanks between Ken-C8 and N53-2. *BnaA09G0559300ZS* encodes an AUXIN RESPONSE FACTOR 18 (ARF18) protein, which has been proven to negatively affect the seed weight and silique length in both *B. napus* and *Arabidopsis* [35]. In this study, *BnaA09G0559300ZS* was located in the ChrA09 QTL hotspot, and the expression of *BnaA09G0559300ZS* in BP was consistently lower than in LP and SP, which is consistent with their phenotype variation. Additionally, 56 SNPs and 19 Indels were detected within *BnaA09G0559300ZS* and its 2 kb flanks between Ken-C8 and N53-2. *BnaC01G0103100ZS* encodes a NAC family TF KIR1, known as a positive regulator of programmed cell death [36], while in the present study, *BnaC01G0103100ZS* was co-related with 62 PDRs and highly expressed in LP and SP compared to BP. Apart from that, *BnaC01G0103100ZS* was located in the ChrC01 QTL hotspot. Six SNPs and three Indels were also detected within *BnaC01G0103100ZS* and its 2-kb flanks between Ken-C8 and N53-2. *BnaA09G0560900ZS* was also within the ChrA09 QTL hotspot and encodes a B3 TF homologous to AtNGA2, a known negative regulator of cell proliferation. In addition, 39 SNPs and 12 Indels were detected within *BnaA09G0560900ZS* and its 2-kb flanks between Ken-C8 and N53-2. In *Arabidopsis*, the overexpression of all four *NGAs* (*AtNGA1* ~ *AtNGA4*) resulted in small and narrow lateral organs, while their knockout produced large and wide lateral organs [37]. *BnaC09G0128900ZS* homology with *PRF1*, whose mutation would cause elongated hypocotyl and root hair phenotypes in *Arabidopsis* [38]*. BnaA09G0543400ZS* was also within the ChrA09 QTL hotspot and is homologous to *At.VLN4*, a major actin filament bundling protein involved in root hair growth by regulating actin organization in a Ca^2+^-dependent manner [39]. However, there was no report of *PRF1* or *VLN4* controlling petal morphology.

**Fig. 7:**
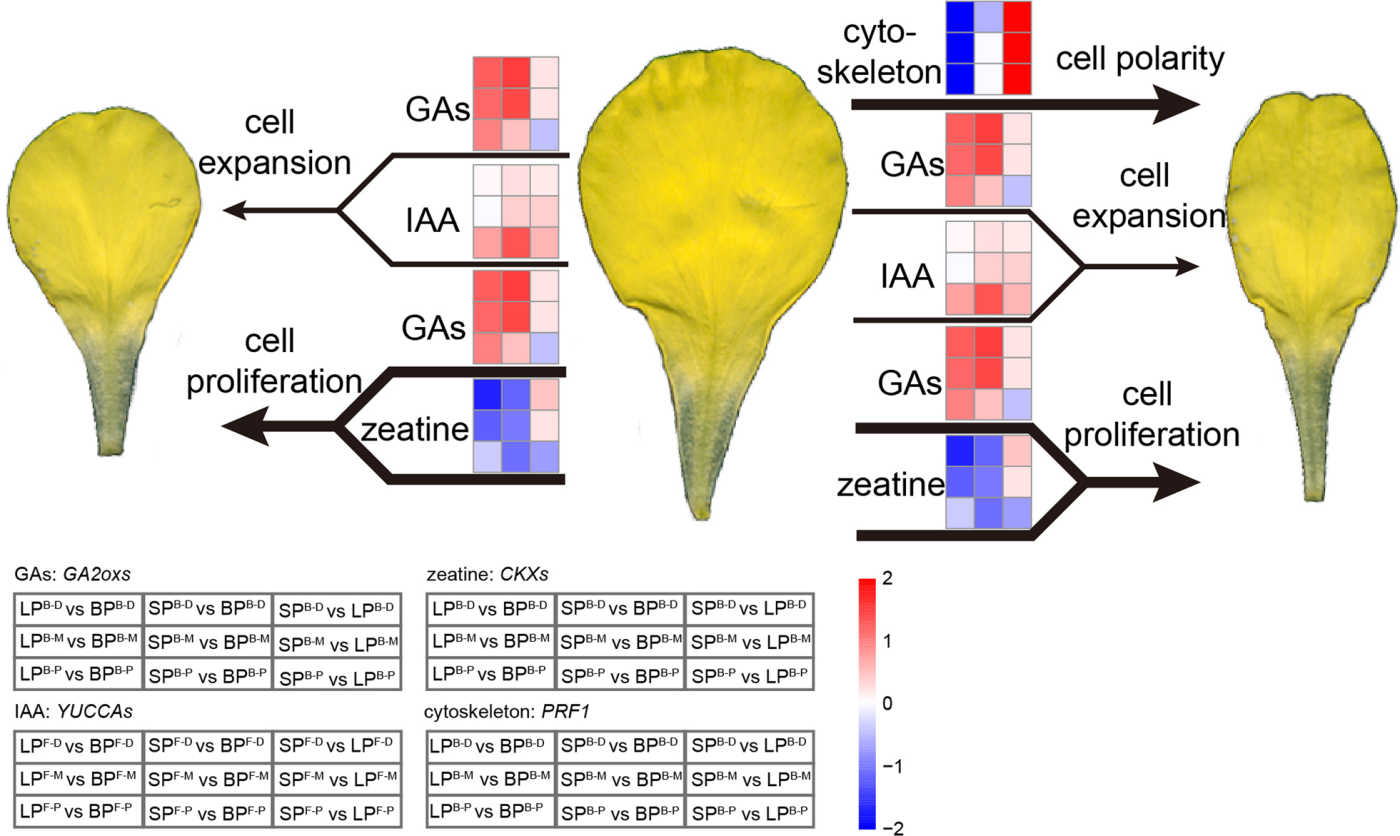
The model of *B. napus* petal morphology variation. The accumulation of GAs and zeatin could promote the cell proliferation of petals, and the accumulation of GAs and IAA could promote the cell expansion of petals, resulting in petal size variation. Meanwhile, the cytoskeleton could affect the cell polarity of division and expansion, potentially leading to slender petals. The heat map was drawn using the log2(fold change) of FPKM of certain genes between specific samples that were detailedly annotated at the bottom, and the matchup of heat map cubes and samples was also annotated at the bottom

Finally, a regulation model of *B. napus* petal morphology was constructed, in which cell proliferation induced by GAs and zeatin was the main factor controlling petal morphology, and the role of cell expansion induced by IAA was also assignable. Meanwhile, a notable effect of cytoskeleton on LP formation was observed (Fig. 7).

## Discussion

The petal morphology of *B. napus* is an essential factor for both horticulture value and crop yield, which has been a research hotspot in recent years [3]. In previous studies, an approximately normal distribution and high heritability (around 60%) of petal size have been consistently detected in different *B. napus* populations [19], while they were focused only on the petal size variation in natural populations. In the present study, six parameters of petal morphology were measured in the KN DH population comprising 300 lines. Dramatic variations of all six parameters were observed, as well as approximately normal distributions and significant transgressive segregation, which is consistent with previous studies in *B. napus* and other flowers (such as *B. oleracea* [40], *B. rapa* [41], *P. axillaris*, and *P. integrifolia* [42]), and indicated that petal morphology is a quantitative trait that controlled by numbers of micro-effect loci. Apart from that, higher broad-sense heritabilities were detected in the present study (over 70%), and significant petal morphology variations between winter and semi-winter environments were reported for the first time. It is also the first time petal size and shape were investigated in the *B. napus* DH population.

QTL mapping, including linkage mapping and GWAS, has proven to be an effective strategy for quantitative trait analysis. In previous studies, Qian et al. identified 16 significant SNPs associated with petal size in a *B. napus* natural population comprising 588 accessions over three microenvironments, most of which were located on ChrA05 and ChrC06 [19]. Wang et al. identified 102 and 49 significant SNPs associated with petal size in a natural population with 295 *B. napus* accessions in two microenvironments, respectively, among which 20 SNPs could be consistently detected in both microenvironments [3]. In the present study, 243 identified QTLs and 211 consensus QTLs contributing to six petal morphology traits were identified in six different microenvironments. Meta-analysis revealed 28 environmentally stable consensus QTLs and 58 unique QTLs control more than one trait. In addition, five QTL hotspots located on ChrA03, ChrA06, ChrA09, ChrC01, and ChrC06 were observed. Among them, 11 identified QTLs were co-located with 25 significant loci previously reported. For example, *qMPP-19YL16-3*, located in the ChrC06 QTL hotspot, was co-located with SNP marker *Bna-C06-p30332388*, which was previously reported to be significantly associated with petal size [19]. Four QTLs in the ChrA09 QTL hotspot (*qPCD-18YL9-3*, *qPAR-18YL9-3*, *qPCD-18YL9-2*, *qPAR-18WH9-2*) were co-located with the SNP marker *Bna-A09-p31953242*, which was also previously reported significantly associated with petal size [19]. Moreover, *qMPL-18WH4-1* was co-located with two SNPs (*A4_660546* and *A4_660552*) that significantly associated with petal size [3] (Additional file 2: Table S18). These results proved the accuracy of our QTL mapping, and the other 232 identified QTLs and 3 QTL hotspots were novel.

Plant organ morphology was determined by cell number and cell size, which are controlled by cell proliferation and cell expansion, respectively [4, 43]. Cytokinin is an effective plant hormone that promotes cell proliferation during the early stage of organ growth [44], while auxin is the most critical plant hormone that controls cell expansion during the later stage of organ growth [45]. Additionally, GAs contribute to both cell proliferation and cell expansion [46]. Over-accumulated or blocked signals of those plant hormones can lead to significant variations in petal morphology. For instance, the MYB73/TPL/HDA19-miR159-CKX6 module controls petal size in *Rosa hybrid* by regulating cytokinin catabolism [4]. Application of exogenous GAs can significantly increase the elongation rate of petal tissue in the *Gerbera hybrid* [47]. A previous study also demonstrated that cytokinin could increase petal size by increasing cell number in *B. napus* [19]. In the present study, the morphology and number of the petal epidermal cells were investigated, and variations in both cell number and cell size between petals with different morphologies were observed, with cell number having a larger contribution to petal morphology than cell size. The spatiotemporal transcriptomic analysis revealed that, apart from cytokinin, auxin and GAs were also important petal morphology regulators in *B. napus*.

In addition to its essential role in cell division [48], the cytoskeleton is crucial for cell wall synthesis and cell polarity establishment, thereby controlling plant organ morphology [49–51]. For example, SPK1 has been found to promote the isotropic organization of cortical MT arrays, thereby inhibiting anisotropic growth in petals [17]. The MT-associated protein IPGA1 regulates the petal morphology by affecting cortical MT organization, and the mutation of *IPGA1* causes an elongated petal phenotype [52]. In maize, ZmLNG1 functions as an organ-shape regulator by affecting the assembly of the TON1-TRM-PP2A complex and subsequently affecting the preprophase band formed by parallel MTs [53]. In the present study, GO terms related to the cytoskeleton, such as “protein phosphatase type 2A complex” and “cytoskeletal protein binding” were significantly enriched. Furthermore, nine cytoskeletal protein binding genes displayed consistent expression variation between elongated and rounded petals were identified, which indicated that the cytoskeleton may also affect the petal morphology by modulating the cell division and polar expansion of petal epidermal cells in *B. napus*.

## Conclusions

QTL mapping of the six petal morphology parameters (including MPA, MPP, MPL, MPW, PAR, and PCD) identified 232 novel QTLs and three novel QTL hotspots in this study. By combining QTL mapping and RNA-seq, a model of petal morphology variation was presented. Specifically, the accumulation of GAs and zeatin could promote the cell proliferation of petals, and the accumulation of GAs and IAA could promote the cell expansion of petals, leading to variations in petal size. Meanwhile, the cytoskeleton could affect the cell polarity of division and expansion, potentially result in slender petals. Subsequently, 61 high-confidence candidate genes, including 30 previously reported PDRs and 31 novel genes, were selected as potential regulators of *B. napus* petal morphology, such as *BnaA06.SAUR10* and *BnaC01.KIR1*. Our present study provided new loci and sights for breeding *B. napus* with desirable petal morphologies.

### Supplementary Information

**Additional file 1: Fig. S1.** The frequency histograms of MPA (**A**), MPP (**B**), PAR (**C**), MPL (**D**), MPW (**E**), and PCD (**F**) in 17WH microenvironment. The frequency histograms of MPA (**G**), MPP (**H**), PAR (**I**), MPL (**J**), MPW (**K**), and PCD (**L**) in 17YL microenvironment. **Fig. S2.** The frequency histograms of MPA (**A**), MPP (**B**), PAR (**C**), MPL (**D**), MPW (**E**), and PCD (**F**) in 18WH microenvironment. The frequency histograms of MPA (**G**), MPP (**H**), PAR (**I**), MPL (**J**), MPW (**K**), and PCD (**L**) in 18YL microenvironment. **Fig. S3.** The frequency histograms of MPA (**A**), MPP (**B**), PAR (**C**), MPL (**D**), MPW (**E**), and PCD (**F**) in 19WH microenvironment. The frequency histograms of MPA (**G**), MPP (**H**), PAR (**I**), MPL (**J**), MPW (**K**), and PCD (**L**) in 19YL microenvironment. **Fig. S4.** All identified QTLs detected in this study controlled MPA, MPP, PAR, MPL, MPW, and PCD in the KN DH population. **Fig. S5.** The PCD variations of lines with different genetic backgrounds in the ChrA03 (**A**), ChrA06 (**B**), ChrA09 (**C**), ChrC01 (**D**), and ChrC06 (**E**) QTL hotspots in all six microenvironments, including 17WH, 17YL, 18WH, 18YL, 19WH, and 19YL. The DH lines that inherited chromosome segments in the specific QTL hotspot from Ken-C8 and N53-2 were annotated “Ken-C8” and “N53-2” in the superscript, respectively. The variant significance was evaluated by the two-tailed one-way ANOVA, and the p-values were annotated above the histograms. **Fig. S6.** The GO (**A**) and KEGG (**B**) enrichment analysis of the 5292 genes within QTL hotspots. **Fig. S7.** The GO (**A**) and KEGG (**B**) enrichment analysis of all genes within the QTL CIs. **Fig. S8. A** The STM images of the epidermis cell of different petals and parts in the flower stage (annotated in the upper right corner). **B** The area (left y-axis) and estimated epidermis cell number (right y-axis) of different petals and parts in the flower stage. The significance of area and cell number variations of the entire petal between diverse forms of petals were verified by two-tailed one-way ANOVA, respectively, and the p-values were annotated above the histograms. **C** The epidermis cell width of MC and ME parts of diverse forms of petals. **Fig. S9.** The epidermis cell area of the DC and DE (**A**), MC and ME (**B**), PC and PE (**C**) part of diverse forms of petals in the bud and flower stages. As well as the epidermis cell length of the DC and DE (**D**), MC and ME (**E**), PC and PE (**F**) of diverse forms of petals in the bud and flower stages. **Fig. S10. A** The schematic diagram of the distal (upper red frames), middle (middle red frames), and proximal (lower red frames) parts of petals in the bud stage (right) and the flower stage (left). **B** The image of the intact booms (bud stage) and flowers (flower stage) of BP (left), SP (middle), and LP (right), and the red bar represents 10 mm. **Fig. S11.** The dendrogram of all RNA-seq samples based on their gene expression profile. **Fig. S12. A** The heat map of the expression level of the seven randomly selected genes in RNA-seq result, which was drawn using log_10_(FPKM). **B** The heat map of the expression level of the seven randomly selected genes measured by qRT-PCR, which was drawn using log2(-△△t). **Fig. S13.** The Venn diagram of up-regulated DEGs of BP^B−D^ vs LP^B−D^, BP^B−D^ vs SP^B−D^, BP^B−M^ vs LP^B−M^, BP^B−M^ vs SP^B−M^, BP^B−P^ vs LP^B−P^, and BP^B−P^ vs SP^B−P^ (**A**). The Venn diagram of down-regulated DEGs of BP^B−D^ vs LP^B−D^, BP^B−D^ vs SP^B−D^, BP^B−M^ vs LP^B−M^, BP^B−M^ vs SP^B−M^, BP^B−P^ vs LP^B−P^, and BP^B−P^ vs SP^B−P^(**B**). The Venn diagram of up-regulated DEGs of BP^F−D^ vs LP^F−D^, BP^F−D^ vs SP^F−D^, BP^F−M^ vs LP^F−M^, BP^F−M^ vs SP^F−M^, BP^F−P^ vs LP^F−P^, and BP^F−P^ vs SP^F−P^ (**C**). The Venn diagram of down-regulated DEGs of BP^F−D^ vs LP^F−D^, BP^F−D^ vs SP^F−D^, BP^F−M^ vs LP^F−M^, BP^F−M^ vs SP^F−M^, BP^F−P^ vs LP^F−P^, and BP^F−P^ vs SP^F−P^ (**D**). **Fig. S14. A** The Venn diagram of “BP vs LP-up”, “BP vs LP-down”, “BP vs SP-up”, and “BP vs SP-down”. Among them, “BP vs LP-up” represents the common up-regulated DEGs of “BP^B−D^ vs LP^B−D^”, “BP^B−M^ vs LP^B−M^”, “BP^B−P^ vs LP^B−P^”, “BP^F−D^ vs LP^F−D^”, “BP^F−M^ vs LP^F−M^”, and “BP^F−P^ vs LP^F−P^”; “BP vs LP-down” represents the common down-regulated DEGs of “BP^B−D^ vs LP^B−D^”, “BP^B−M^ vs LP^B−M^”, “BP^B−P^ vs LP^B−P^”, “BP^F−D^ vs LP^F−D^”, “BP^F−M^ vs LP^F−M^”, and “BP^F−P^ vs LP^F−P^”; “BP vs SP-up” represents the common up-regulated DEGs of “BP^B−D^ vs SP^B−D^”, “BP^B−M^ vs SP^B−M^”, “BP^B−P^ vs SP^B−P^”, “BP^F−D^ vs SP^F−D^”, “BP^F−M^ vs SP^F−M^”, and “BP^F−P^ vs SP^F−P^”; “BP vs SP-down” represents the common down-regulated DEGs of “BP^B−D^ vs SP^B−D^”, “BP^B−M^ vs SP^B−M^”, “BP^B−P^ vs SP^B−P^”, “BP^F−D^ vs SP^F−D^”, “BP^F−M^ vs SP^F−M^”, and “BP^F−P^ vs SP^F−P^”. **B** The GO enrichment analysis of common DEGs that consistently detected between BP and LP on the three parts and two stages, while the common DEGs consistently detected between BP and SP on the three parts and two stages were excluded meanwhile. Specifically, the up-regulated DEGs processed to GO enrichment analysis were annotated in black color in Figure A, and the down-regulated DEGs processed to GO enrichment analysis were annotated in white color in Figure A. **Fig. S15.** The heat maps of the expression of DNA polymerase complexes genes, which were drawn using log2(fold change) of the FPKM of LP^B−D^ vs BP^B−D^ (**A**), LP^B−P^ vs BP^B−P^ (**B**), LP^B−M^ vs BP^B−M^ (**C**), SP^B−D^ vs BP^B−D^ (**D**), SP^B−P^ vs BP^B−P^ (**E**), and SP^B−M^ vs BP^B−M^ (**F**). **Fig. S16.** The FPKM of *Bn.GA20oxs* in the bud stage petals (**A**), *Bn.GA3oxs* in the bud stage petals (**B**), *Bn.CKXs* in the bud stage petals (**C**), and *Bn.YUCCAs* in flower stage petals (**D**). **E** The heat map of KEGG orthology that participated in the auxin signal transduction, which was drawn using log2(fold change) of the FPKM. The matchup of heat map cubes and samples were annotated at the bottom. **Fig. S17. A** The distribution of all TFs (above the x-axis) and TFs correlated with PDRs (under the x-axis) within the five QTL hotspots that belong to different TF families. **B** The heat map of the expression of 19 high-confidence candidate TF genes that were expressed in petals, which was drawn using their FPKMs that normalized by z-score.

**Additional file 2: Table S1.** The statistical data of MPA, MPP, PAR, MPL, MPW, and PCD in the KN DH population under six microenvironments, including 17WH, 17YL, 18WH, 18YL, 19WH, and 19YL. **Table S2.** The Pearson correlation coefficient of MPA, MPP, PAR, MPL, MPW, and PCD in 17WH. The p-value was represented by “*” (p < 0.05, significant) and “**” (p < 0.01, highly significant). **Table S3.** The Pearson correlation coefficient of MPA, MPP, PAR, MPL, MPW, and PCD in 17YL. The p-value was represented by “*” (p < 0.05, significant) and “**” (p < 0.01, highly significant). **Table S4.** The Pearson correlation coefficient of MPA, MPP, PAR, MPL, MPW, and PCD in 18WH. The p-value was represented by “*” (p < 0.05, significant) and “**” (p < 0.01, highly significant). **Table S5.** The Pearson correlation coefficient of MPA, MPP, PAR, MPL, MPW, and PCD in 18YL. The p-value was represented by “*” (p < 0.05, significant) and “**” (p < 0.01, highly significant). **Table S6.** The Pearson correlation coefficient of MPA, MPP, PAR, MPL, MPW, and PCD in 19WH. The p-value was represented by “*” (p < 0.05, significant) and “**” (p < 0.01, highly significant). **Table S7.** The Pearson correlation coefficient of MPA, MPP, PAR, MPL, MPW, and PCD in 19YL. The p-value was represented by “*” (p < 0.05, significant) and “**” (p < 0.01, highly significant). **Table S8.** The detailed information of all identified QTLs detected in the present study. The “position”, “from”, and “to” were presented by the genetic distance. **Table S9.** The detailed information of all consensus QTLs detected in the present study, and the “position”, “from”, and “to” were presented by the genetic distance. **Table S10.** The detailed information of all unique QTLs detected in the present study, and the “position”, “from”, and “to” were presented by the genetic distance. **Table S11.** All PDRs collected in the present study. **Table S12.** The detailed information of each RNA-seq sample. **Table S13.** The primers used in the present study. **Table S14.** The detailed information of candidate genes and high-confidence candidate genes. **Table S15.** The sequence variations of candidate genes. **Table S16**. The expression profile (presented by FPKM) of 152 candidate PDRs expressed in the flower stage petals (B ~ J) and their log2(FC) between different petal samples (K ~ P). The zero values of FPKMs were replaced by 0.01 to avoid invalid parameters. **Table S17**. The expression profile (presented by FPKM) of 168 candidate PDRs expressed in the bud stage petals (B ~ J) and their log2(FC) between different petal samples (K ~ P). The zero values of FPKMs were replaced by 0.01 to avoid invalid parameters. **Table S18.** The previously reported petal size QTLs of *B. napus* that are consistent with the petal morphology QTLs detected in the present study.

## Data Availability

The raw data of the spatiotemporal transcriptomic analysis of the different parts of diversiform petals in the present study have been uploaded to the NCBI Sequence Read Archive (SRA, https://www.ncbi.nlm.nih.gov/sra) with the accession number PRJNA1052045.

## Abbreviations

QTL: Quantitative trait locus
GWAS: Genome-wide association study
CI: Confidence interval
PV: Phenotypic variation
MT: Microtubule
qRT-PCR: Quantitative real-time PCR
TIP41: TAP42 INTERACTING PROTEIN OF 41 KDA
DH: Double haploid
WH: Wuhan
YL: Yangling
MPL: Mean petal length
MPW: Mean petal width
MPA: Mean petal area
MPP: Mean petal perimeter
PAR: Petal aspect ratio
PCD: Petal circularity degree
CIM: Composite interval mapping
PDR: Plant organ development regulator
BP: Big round petal
SP: Small round petal
LP: Elongated slender petal
FPKM: Fragments per kilobase of exon model per million mapped fragments
DEG: Differential expressed gene
TF: Transcriptional factor
STM: Scanning tunneling microscope
GA: Gibberellin
IAA: Indoleacetic acid
CKX: Cytokinin oxidase/dehydrogenase
SAUR10: Small auxin upregulated RNA 10
ARF18: Auxin response factor 18
