# Supplementary material for: An integrated QTL and RNA-seq analysis revealed new petal morphology loci in Brassica napus L
Source: Biotechnol Biofuels Bioprod. 2024 Jul 18;17:105. doi: 10.1186/s13068-024-02551-z (PMC11264636; doi:10.1186/s13068-024-02551-z)
Supplement: Supplementary file 1 — Additional file 1: Fig. S1. The frequency histograms of MPA (A), MPP (B), PAR (C), MPL (D), MPW (E), and PCD (F) in 17WH microenvironment. The frequency histograms of MPA (G), MPP (H), PAR (I), MPL (J), MPW (K), and PCD (L) in 17YL microenvironment. Fig. S2. The frequency histograms of MPA (A), MPP (B), PAR (C), MPL (D), MPW (E), and PCD (F) in 18WH microenvironment. The frequency histograms of MPA (G), MPP (H), PAR (I), MPL (J), MPW (K), and PCD (L) in 18YL microenvironment. Fig. S3. The frequency histograms of MPA (A), MPP (B), PAR (C), MPL (D), MPW (E), and PCD (F) in 19WH microenvironment. The frequency histograms of MPA (G), MPP (H), PAR (I), MPL (J), MPW (K), and PCD (L) in 19YL microenvironment. Fig. S4. All identified QTLs detected in this study controlled MPA, MPP, PAR, MPL, MPW, and PCD in the KN DH population. Fig. S5. The PCD variations of lines with different genetic backgrounds in the ChrA03 (A), ChrA06 (B), ChrA09 (C), ChrC01 (D), and ChrC06 (E) QTL hotspots in all six microenvironments, including 17WH, 17YL, 18WH, 18YL, 19WH, and 19YL. The DH lines that inherited chromosome segments in the specific QTL hotspot from Ken-C8 and N53-2 were annotated “Ken-C8” and “N53-2” in the superscript, respectively. The variant significance was evaluated by the two-tailed one-way ANOVA, and the p-values were annotated above the histograms. Fig. S6. The GO (A) and KEGG (B) enrichment analysis of the 5292 genes within QTL hotspots. Fig. S7. The GO (A) and KEGG (B) enrichment analysis of all genes within the QTL CIs. Fig. S8. A The STM images of the epidermis cell of different petals and parts in the flower stage (annotated in the upper right corner). B The area (left y-axis) and estimated epidermis cell number (right y-axis) of different petals and parts in the flower stage. The significance of area and cell number variations of the entire petal between diverse forms of petals were verified by two-tailed one-way ANOVA, respectively, and the p-values were annotated abo [file 13068_2024_2551_MOESM1_ESM.docx]

**
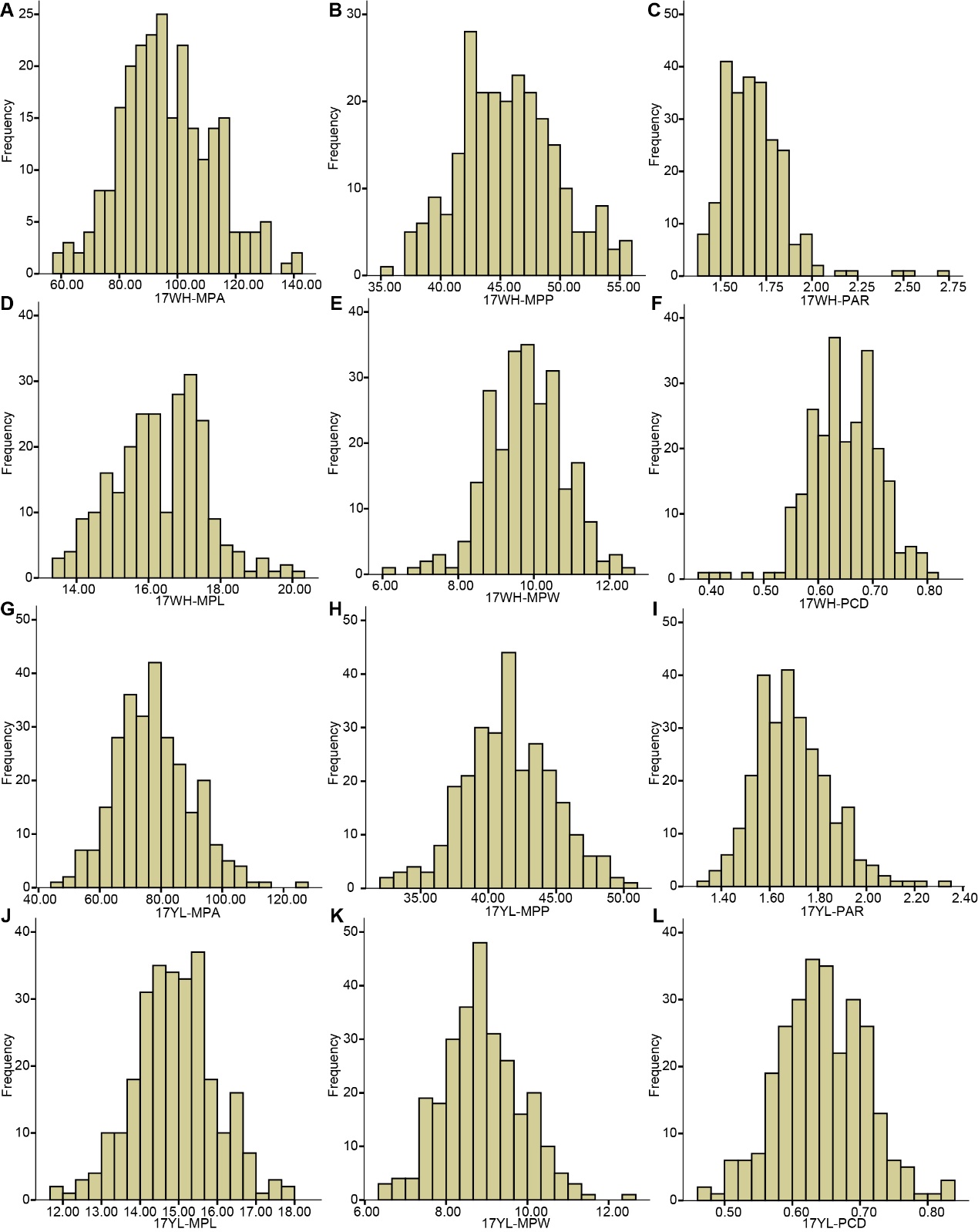
**

**Fig. S1.** The frequency histograms of MPA (**A**), MPP (**B**), PAR (**C**), MPL (**D**), MPW (**E**), and PCD (**F**) in 17WH microenvironment. The frequency histograms of MPA (**G**), MPP (**H**), PAR (**I**), MPL (**J**), MPW (**K**), and PCD (**L**) in 17YL microenvironment.


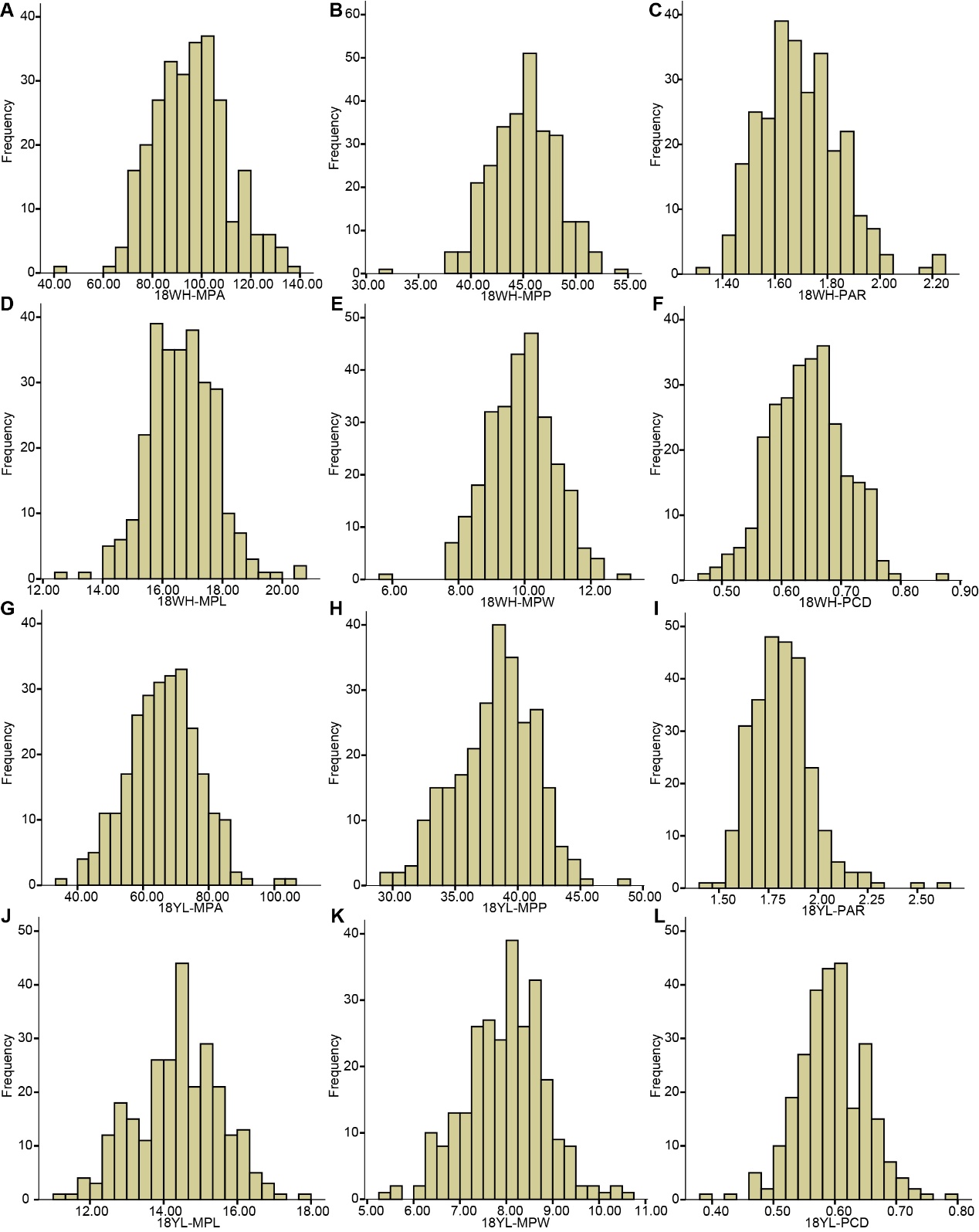


**Fig. S2.** The frequency histograms of MPA (**A**), MPP (**B**), PAR (**C**), MPL (**D**), MPW (**E**), and PCD (**F**) in 18WH microenvironment. The frequency histograms of MPA (**G**), MPP (**H**), PAR (**I**), MPL (**J**), MPW (**K**), and PCD (**L**) in 18YL microenvironment.


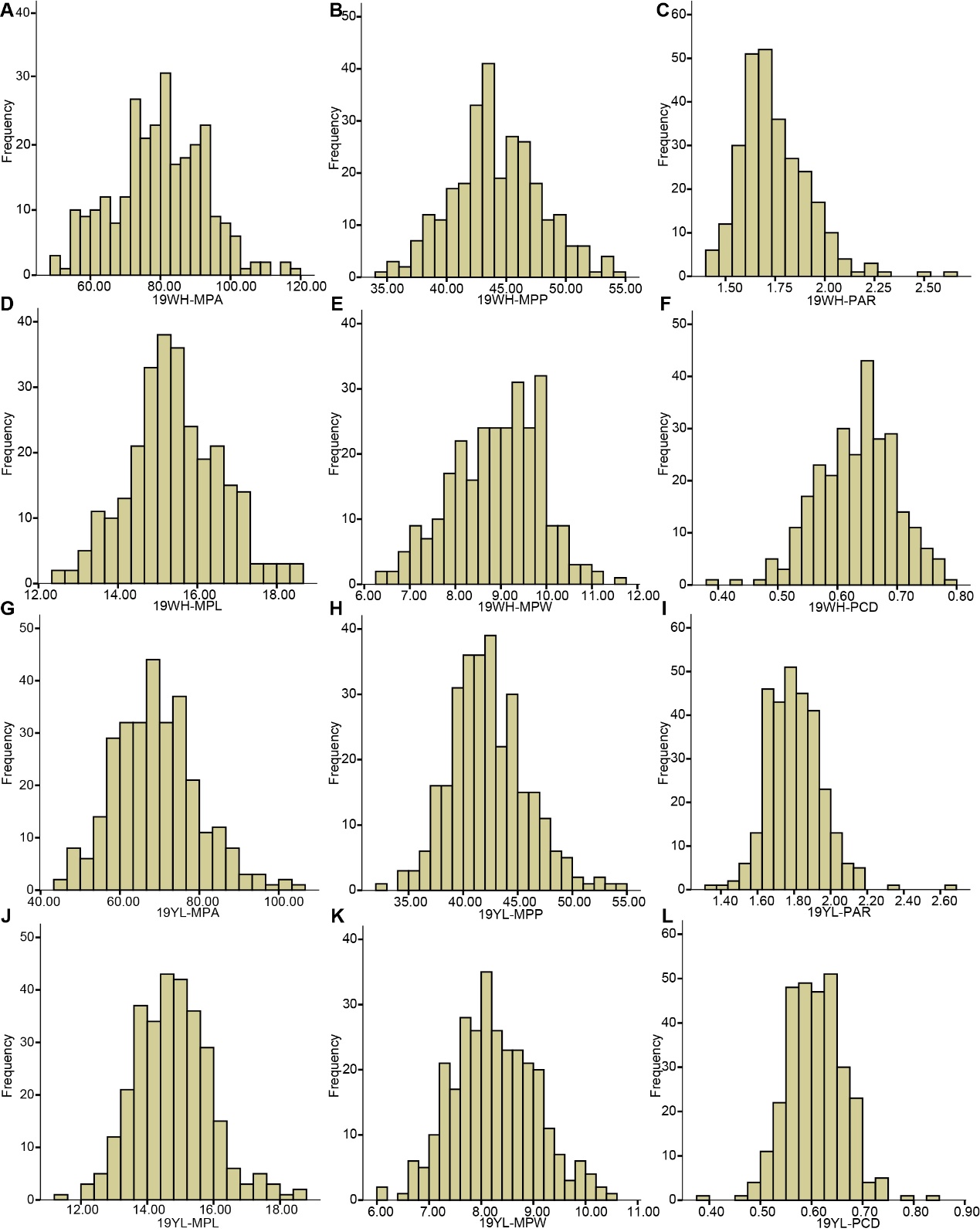


**Fig. S3.** The frequency histograms of MPA (**A**), MPP (**B**), PAR (**C**), MPL (**D**), MPW (**E**), and PCD (**F**) in 19WH microenvironment. The frequency histograms of MPA (**G**), MPP (**H**), PAR (**I**), MPL (**J**), MPW (**K**), and PCD (**L**) in 19YL microenvironment.

**
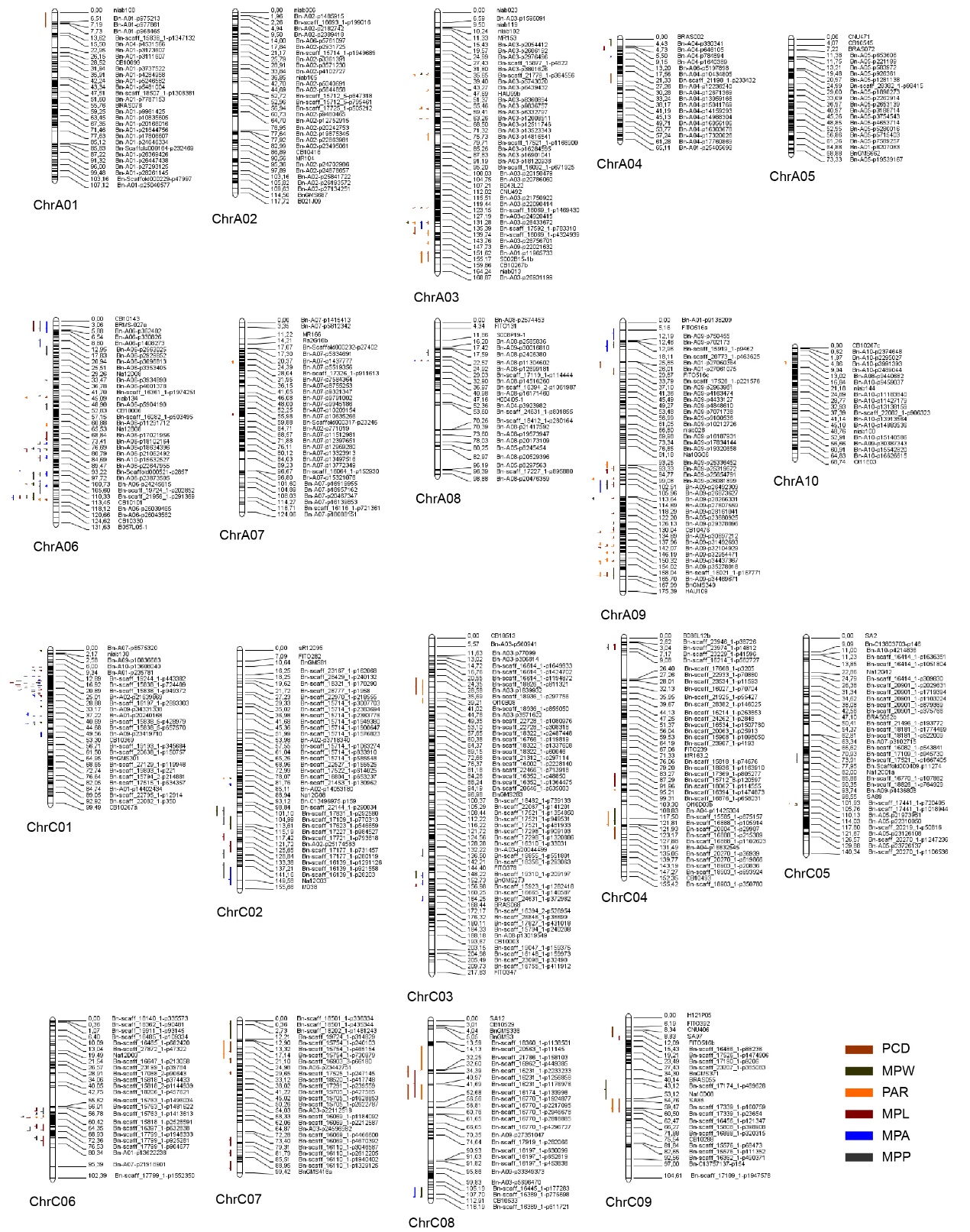
**

**Fig. S4.** All identified QTLs detected in this study controlled MPA, MPP, PAR, MPL, MPW, and PCD in the KN DH population.


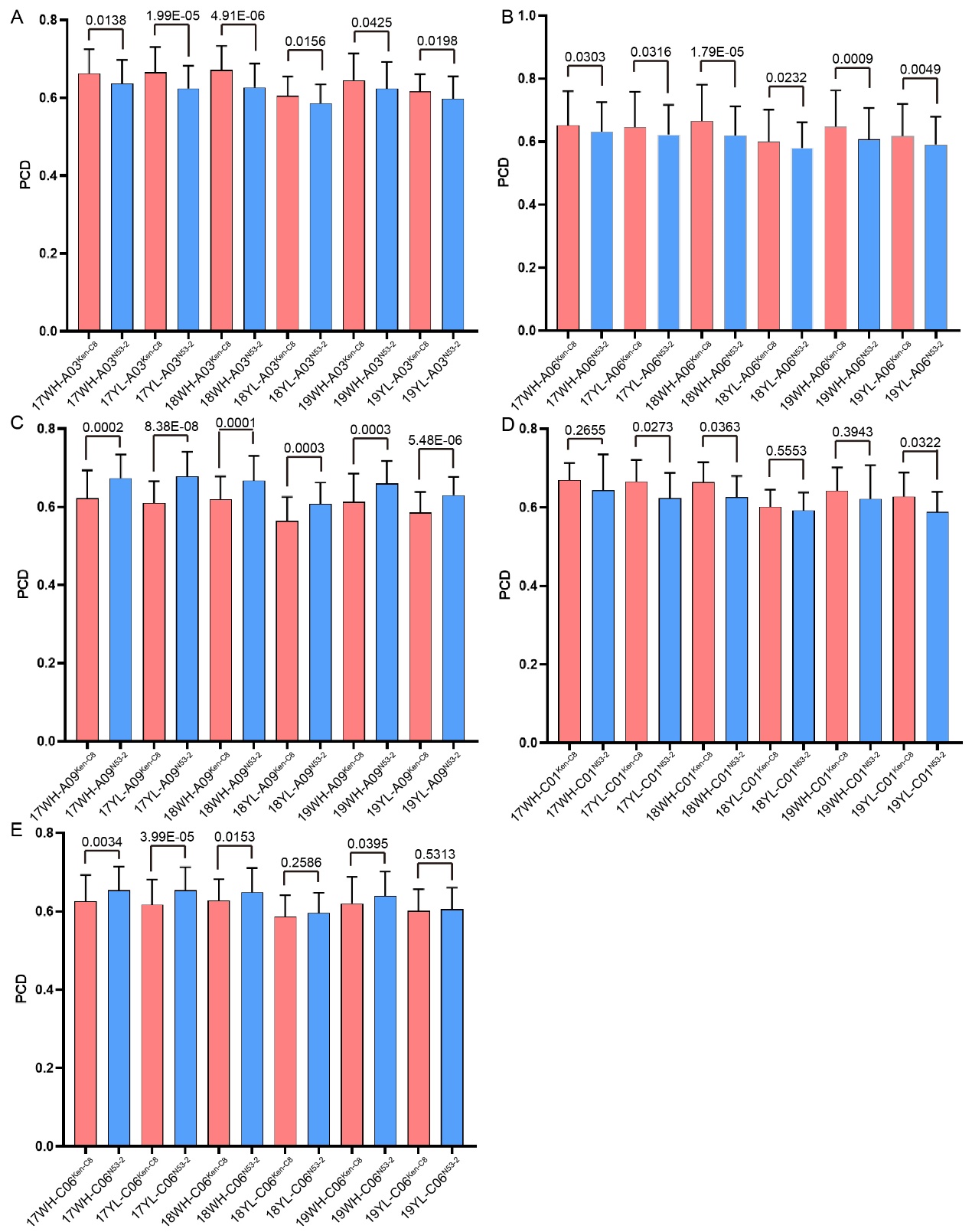


**Fig. S5.** The PCD variations of lines with different genetic backgrounds in the ChrA03 (**A**), ChrA06 (**B**), ChrA09 (**C**), ChrC01 (**D**), and ChrC06 (**E**) QTL hotspots in all six microenvironments, including 17WH, 17YL, 18WH, 18YL, 19WH, and 19YL. The DH lines that inherited chromosome segments in the specific QTL hotspot from Ken-C8 and N53-2 were annotated “Ken-C8” and “N53-2” in the superscript, respectively. The variant significance was evaluated by the two-tailed one-way ANOVA, and the p-values were annotated above the histograms.


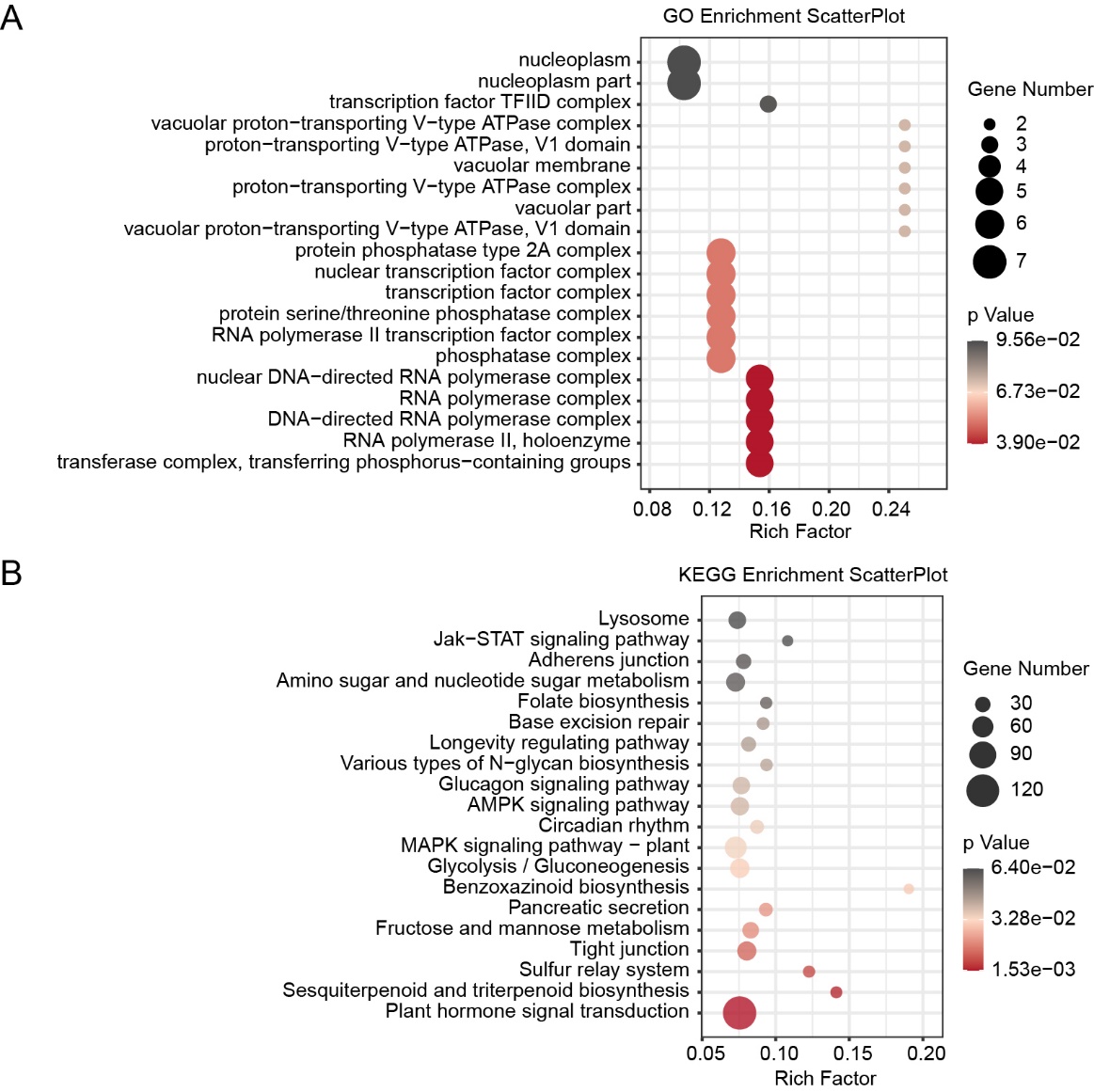


**Fig. S6.** The GO (**A**) and KEGG (**B**) enrichment analysis of the 5292 genes within QTL hotspots.


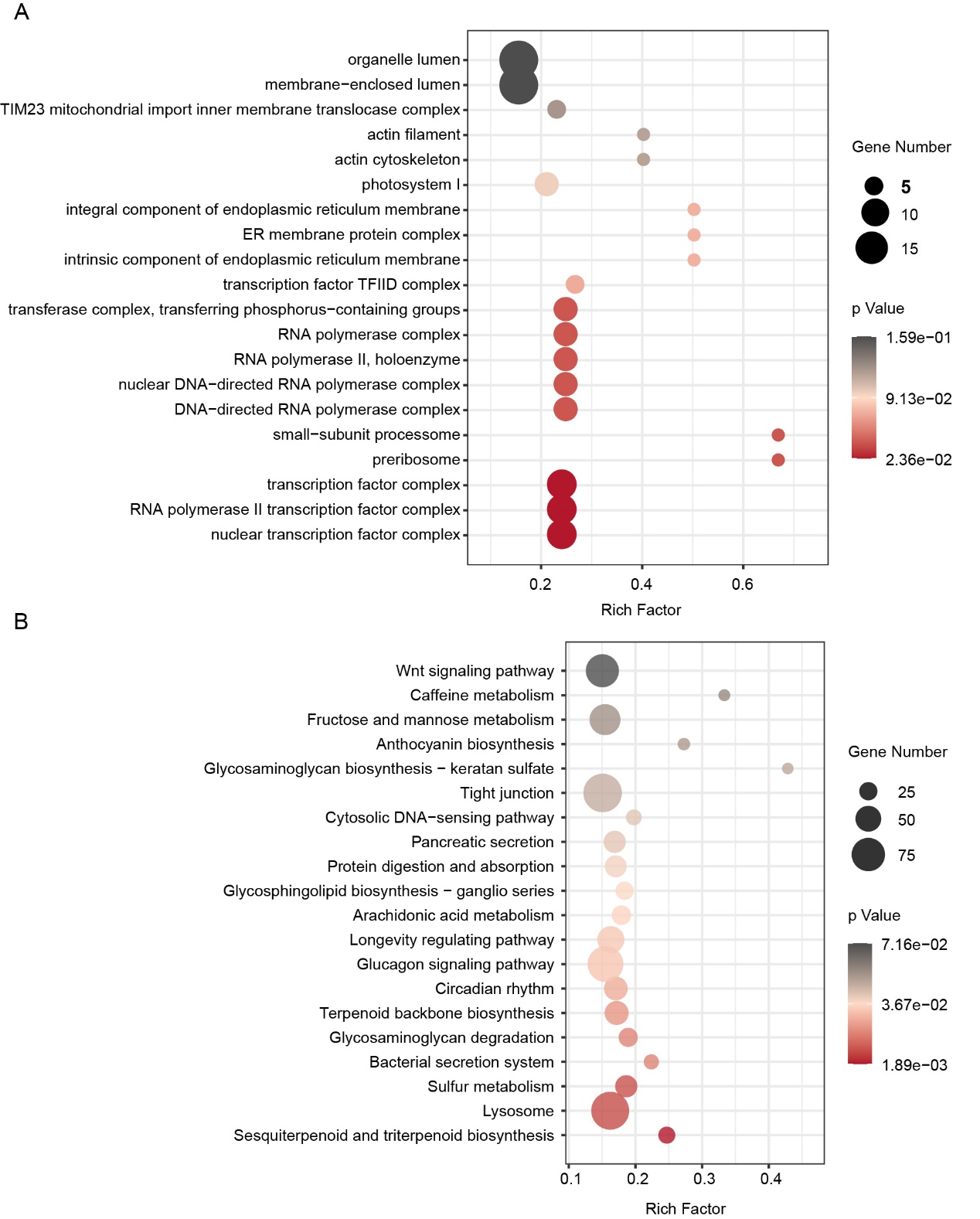


**Fig. S7.** The GO (**A**) and KEGG (**B**) enrichment analysis of all genes within the QTL CIs.


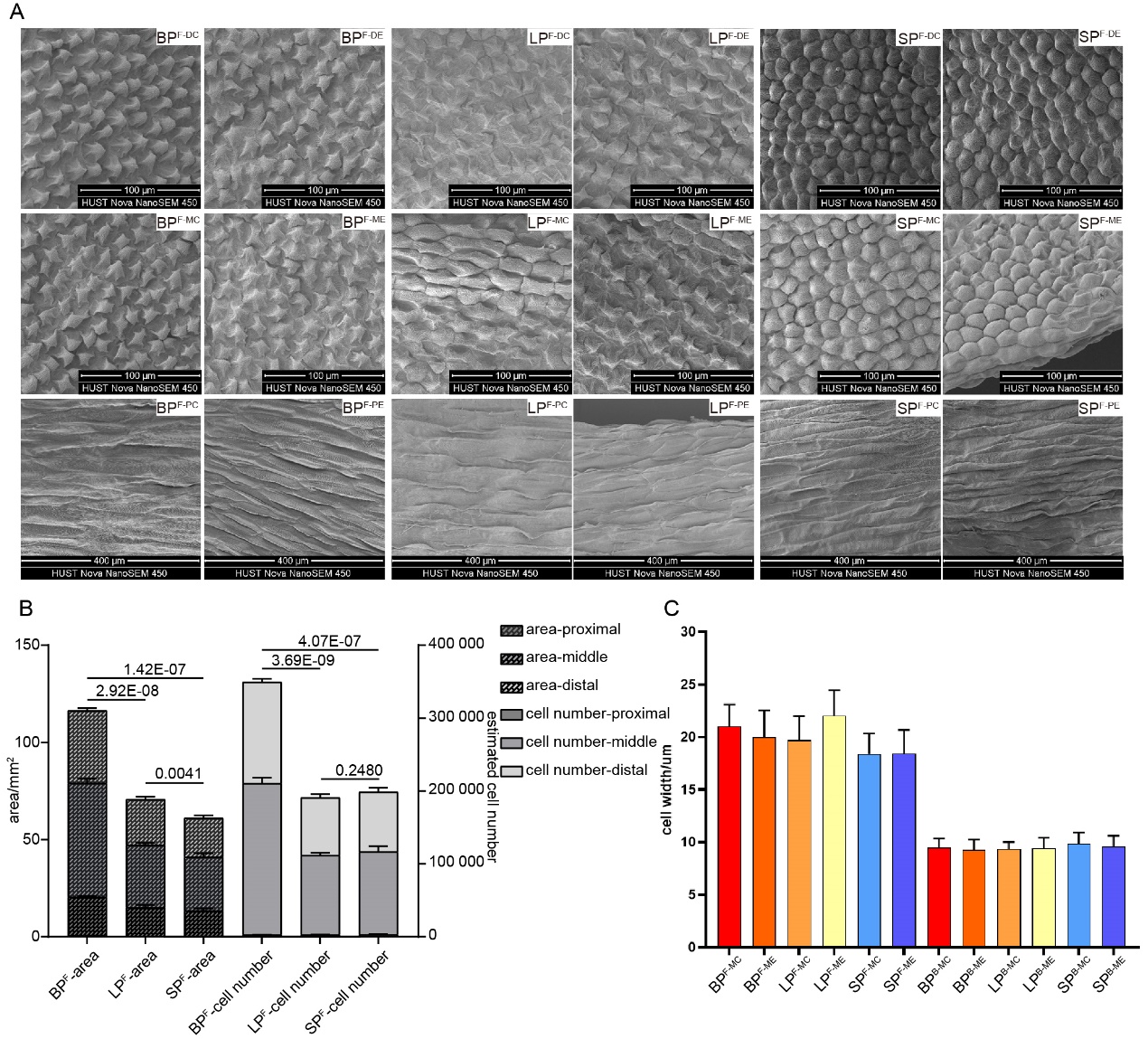


**Fig. S8.** **A** The STM screenage of the epidermis cell of different petals and parts in the flower stage (annotated in the upper right corner). **B** The area (left y-axis) and estimated epidermis cell number (right y-axis) of different petals and parts in the flower stage. The significance of area and cell number variations of the entire petal between diverse forms of petals were verified by two-tailed one-way ANOVA, respectively, and the p-values were annotated above the histograms. **C** The epidermis cell width of MC and ME parts of diverse forms of petals.


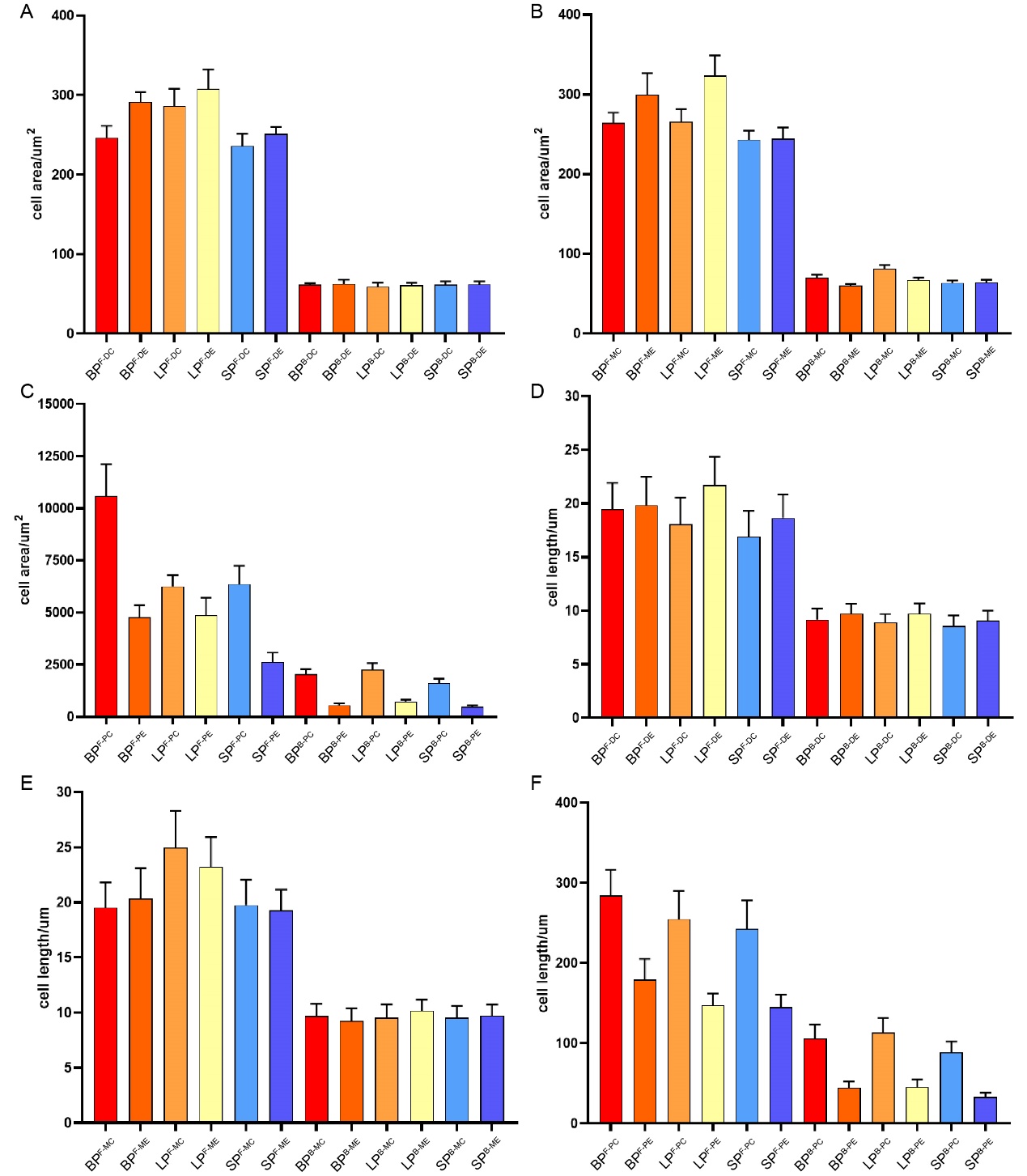


**Fig. S9.** The epidermis cell area of the DC and DE (**A**), MC and ME (**B**), PC and PE (**C**) part of diverse forms of petals in the bud and flower stages. As well as the epidermis cell length of the DC and DE (**D**), MC and ME (**E**), PC and PE (**F**) of diverse forms of petals in the bud and flower stages.


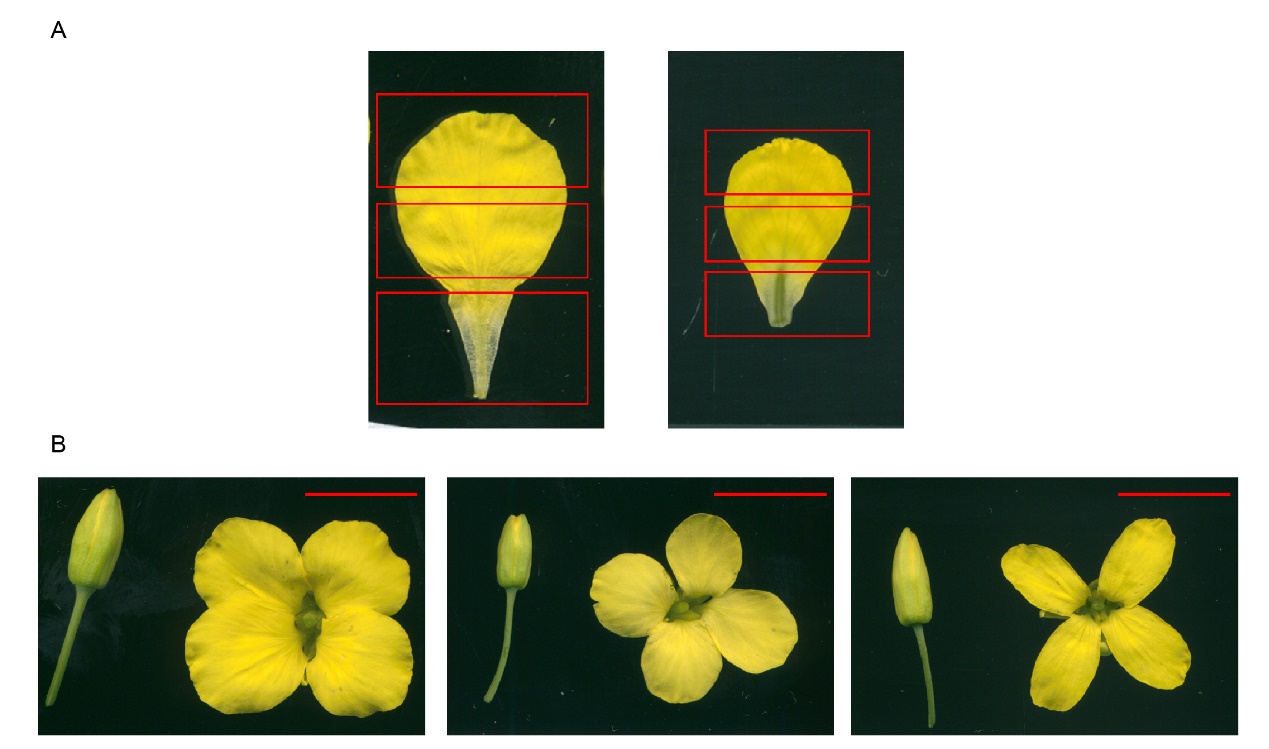


**Fig. S10. A** The schematic diagram of the distal (upper red frames), middle (middle red frames), and proximal (lower red frames) parts of petals in the bud stage (right) and the flower stage (left). **B** The image of the intact booms (bud stage) and flowers (flower stage) of BP (left), SP (middle), and LP (right), and the red bar represents 10 mm.


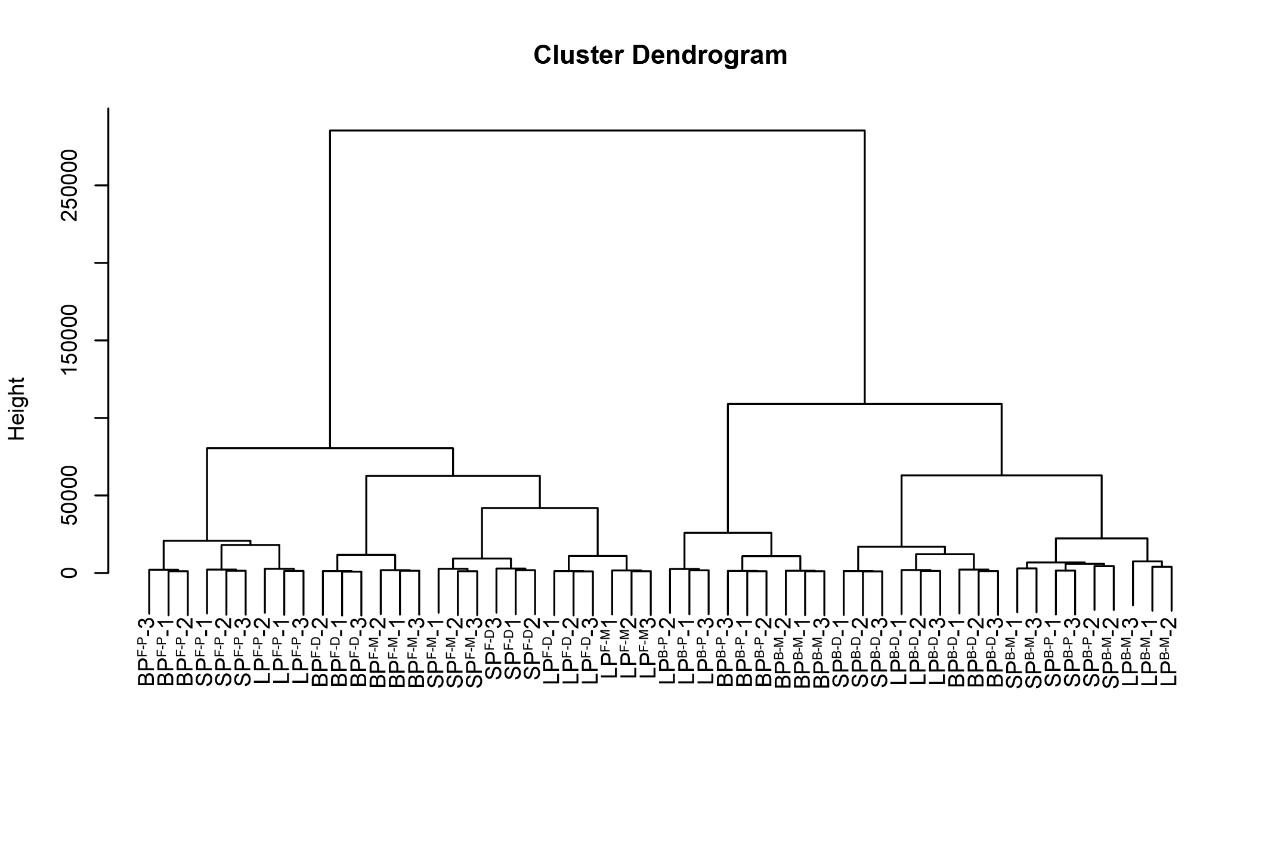


**Fig. S11.** The dendrogram of all RNA-seq samples based on their gene expression profile.


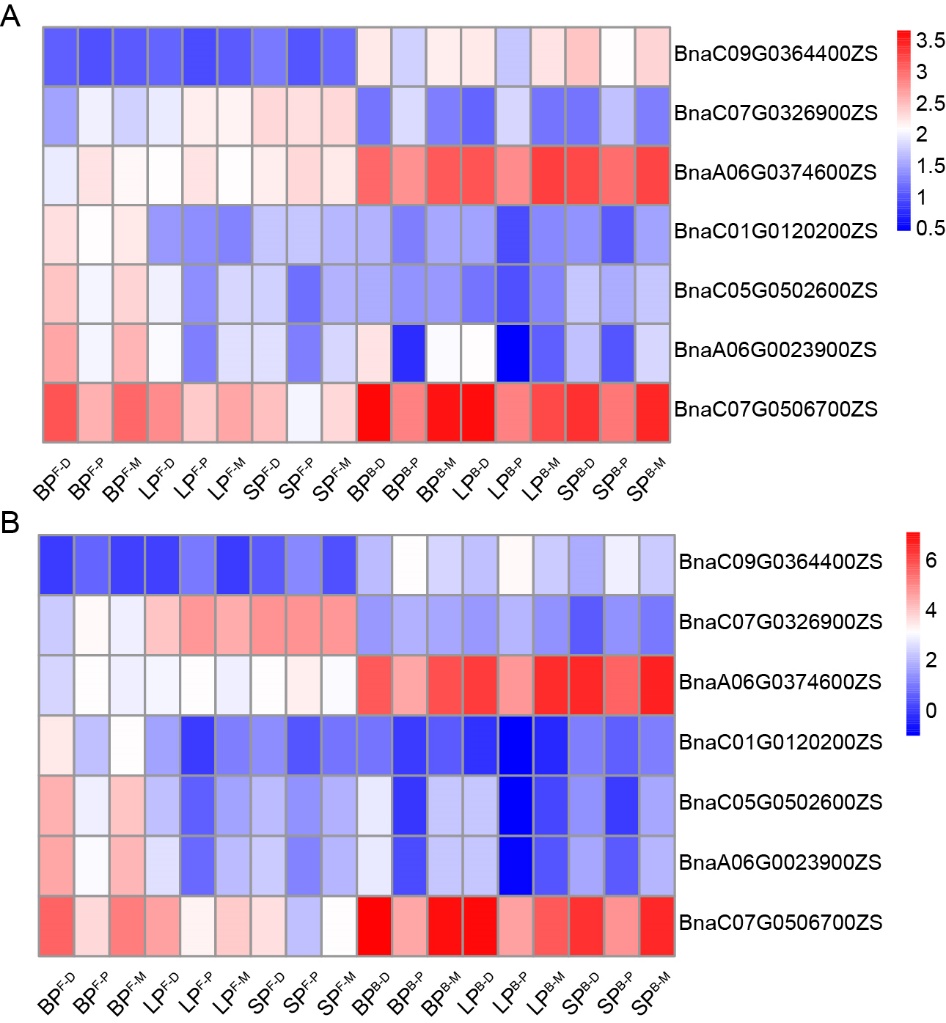


**Fig. S12.** **A** The heat map of the expression level of the seven randomly selected genes in RNA-seq result, which was drawn using log_10_(FPKM). **B** The heat map of the expression level of the seven randomly selected genes measured by qRT-PCR, which was drawn using log2(-△△t).


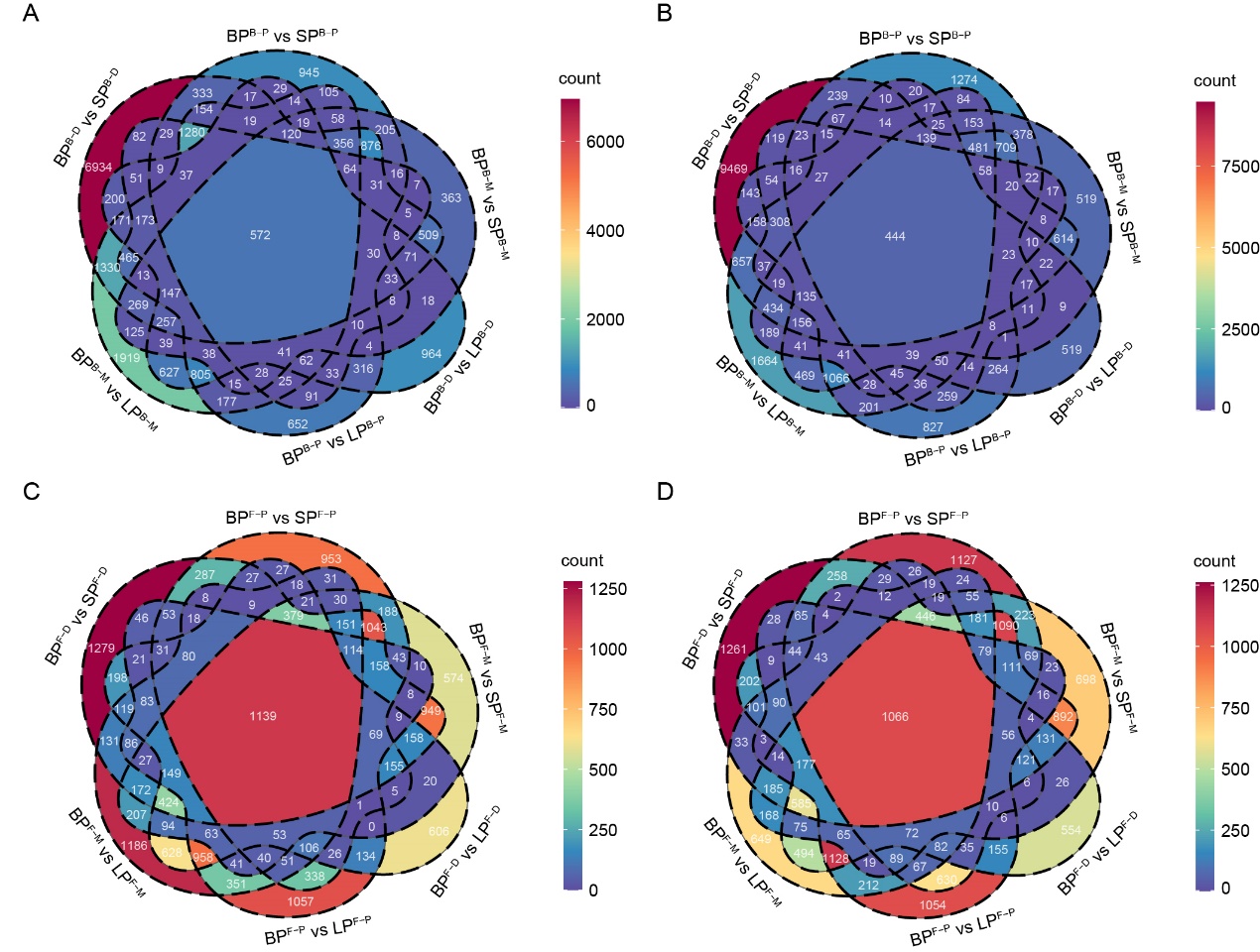


**Fig. S13.** The venn diagram of up-regulated DEGs of BP^B-D^ vs LP^B-D^, BP^B-D^ vs SP^B-D^, BP^B-M^ vs LP^B-M^, BP^B-M^ vs SP^B-M^, BP^B-P^ vs LP^B-P^, and BP^B-P^ vs SP^B-P^ (**A**). The venn diagram of down-regulated DEGs of BP^B-D^ vs LP^B-D^, BP^B-D^ vs SP^B-D^, BP^B-M^ vs LP^B-M^, BP^B-M^ vs SP^B-M^, BP^B-P^ vs LP^B-P^, and BP^B-P^ vs SP^B-P^(**B**). The venn diagram of up-regulated DEGs of BP^F-D^ vs LP^F-D^, BP^F-D^ vs SP^F-D^, BP^F-M^ vs LP^F-M^, BP^F-M^ vs SP^F-M^, BP^F-P^ vs LP^F-P^, and BP^F-P^ vs SP^F-P^ (**C**). The venn diagram of down-regulated DEGs of BP^F-D^ vs LP^F-D^, BP^F-D^ vs SP^F-D^, BP^F-M^ vs LP^F-M^, BP^F-M^ vs SP^F-M^, BP^F-P^ vs LP^F-P^, and BP^F-P^ vs SP^F-P^ (**D**).


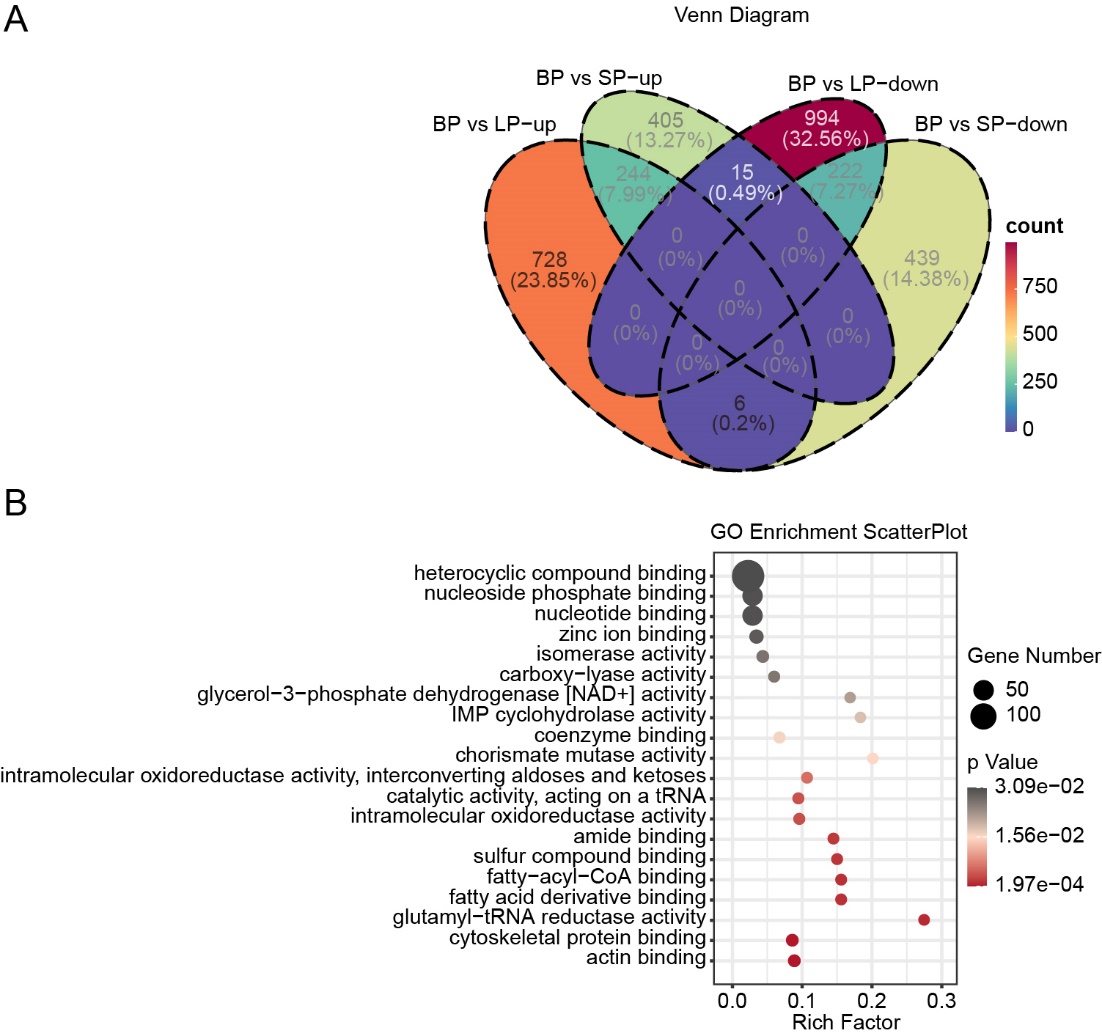


**Fig. S14.** **A** The Venn diagram of “BP vs LP-up”, “BP vs LP-down”, “BP vs SP-up”, and “BP vs SP-down”. Among them, “BP vs LP-up” represents the common up-regulated DEGs of “BP^B-D^ vs LP^B-D^”, “BP^B-M^ vs LP^B-M^”, “BP^B-P^ vs LP^B-P^”, “BP^F-D^ vs LP^F-D^”, “BP^F-M^ vs LP^F-M^”, and “BP^F-P^ vs LP^F-P^”; “BP vs LP-down” represents the common down-regulated DEGs of “BP^B-D^ vs LP^B-D^”, “BP^B-M^ vs LP^B-M^”, “BP^B-P^ vs LP^B-P^”, “BP^F-D^ vs LP^F-D^”, “BP^F-M^ vs LP^F-M^”, and “BP^F-P^ vs LP^F-P^”; “BP vs SP-up” represents the common up-regulated DEGs of “BP^B-D^ vs SP^B-D^”, “BP^B-M^ vs SP^B-M^”, “BP^B-P^ vs SP^B-P^”, “BP^F-D^ vs SP^F-D^”, “BP^F-M^ vs SP^F-M^”, and “BP^F-P^ vs SP^F-P^”; “BP vs SP-down” represents the common down-regulated DEGs of “BP^B-D^ vs SP^B-D^”, “BP^B-M^ vs SP^B-M^”, “BP^B-P^ vs SP^B-P^”, “BP^F-D^ vs SP^F-D^”, “BP^F-M^ vs SP^F-M^”, and “BP^F-P^ vs SP^F-P^”. **B** The GO enrichment analysis of common DEGs that consistently detected between BP and LP on the three parts and two stages, while the common DEGs consistently detected between BP and SP on the three parts and two stages were excluded meanwhile. Specifically, the up-regulated DEGs processed to GO enrichment analysis were annotated in black color in Figure A, and the down-regulated DEGs processed to GO enrichment analysis were annotated in white color in Figure A.


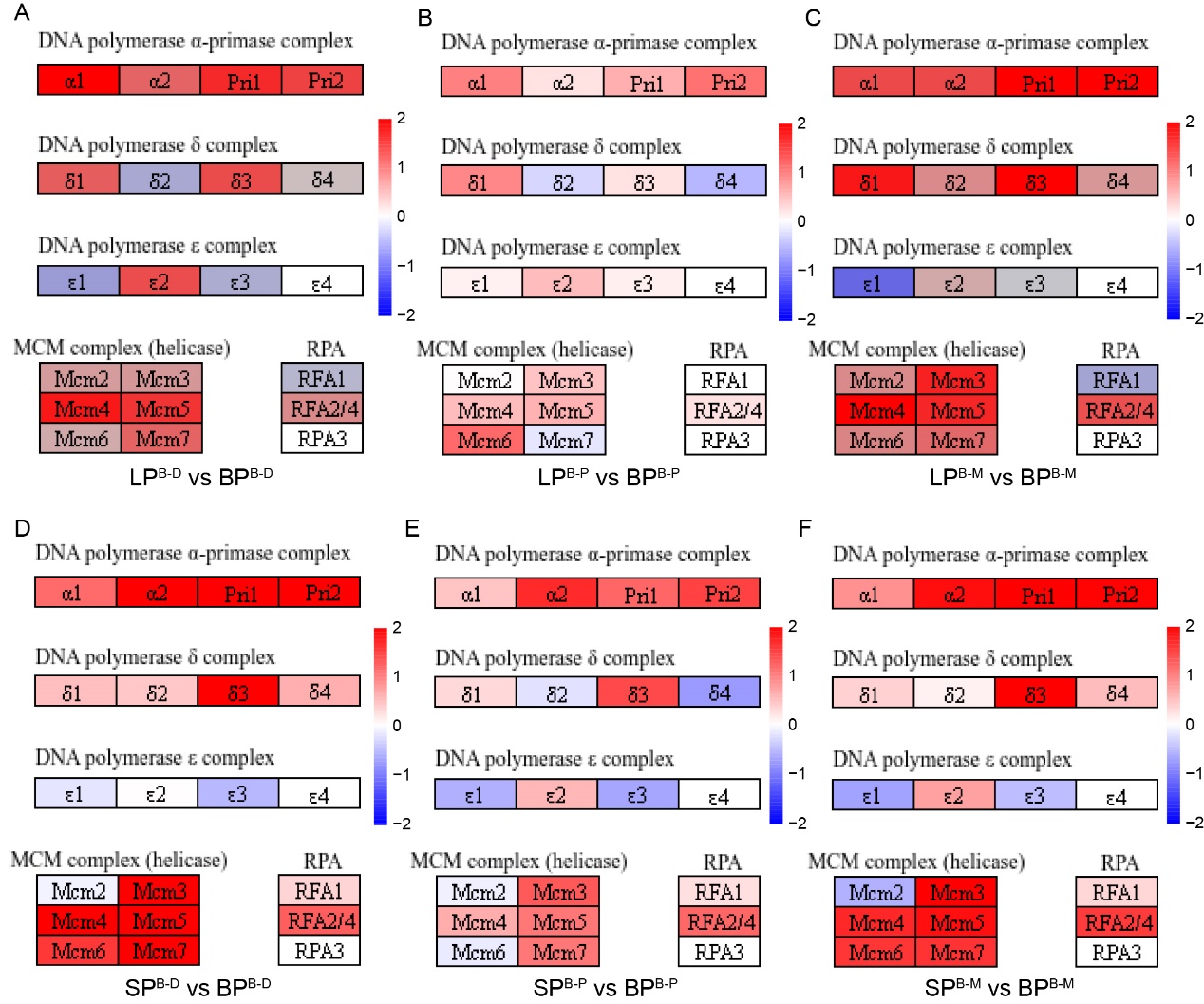


**Fig. S15.** The heat maps of the expression of DNA polymerase complexes genes, which were drawn using log2(fold change) of the FPKM of LP^B-D^ vs BP^B-D^ (**A**), LP^B-P^ vs BP^B-P^ (**B**), LP^B-M^ vs BP^B-M^ (**C**), SP^B-D^ vs BP^B-D^ (**D**), SP^B-P^ vs BP^B-P^ (**E**), and SP^B-M^ vs BP^B-M^ (**F**).


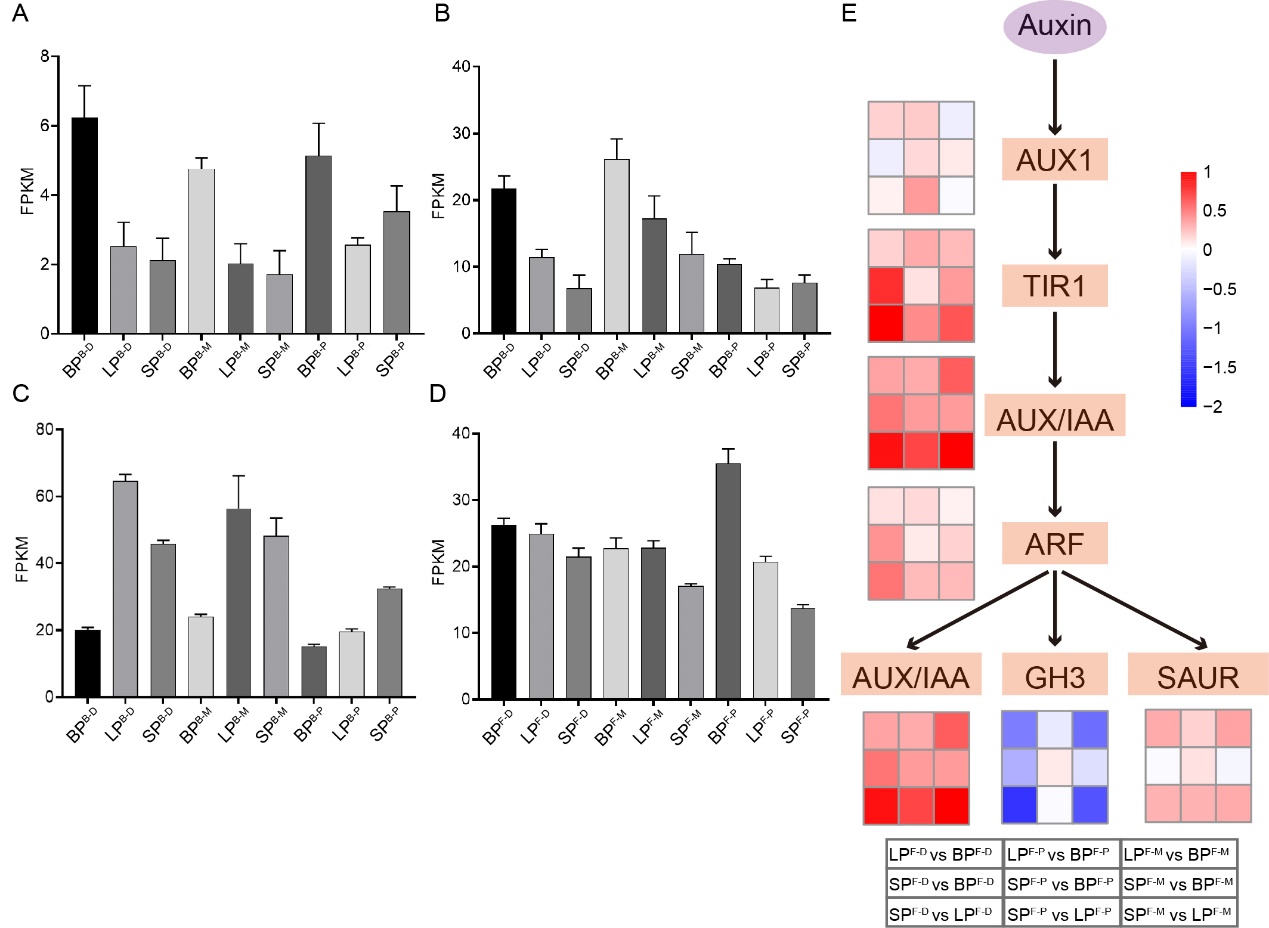


**Fig. S16.** The FPKM of *Bn.GA20oxs* in the bud stage petals (**A**), *Bn.GA3oxs* in the bud stage petals (**B**), *Bn.CKXs* in the bud stage petals (**C**), and *Bn.YUCCAs* in flower stage petals (**D**). **E** The heat map of KEGG orthology that participated in the auxin signal transduction, which was drawn using log2(fold change) of the FPKM. The matchup of heat map cubes and samples were annotated at the bottom.


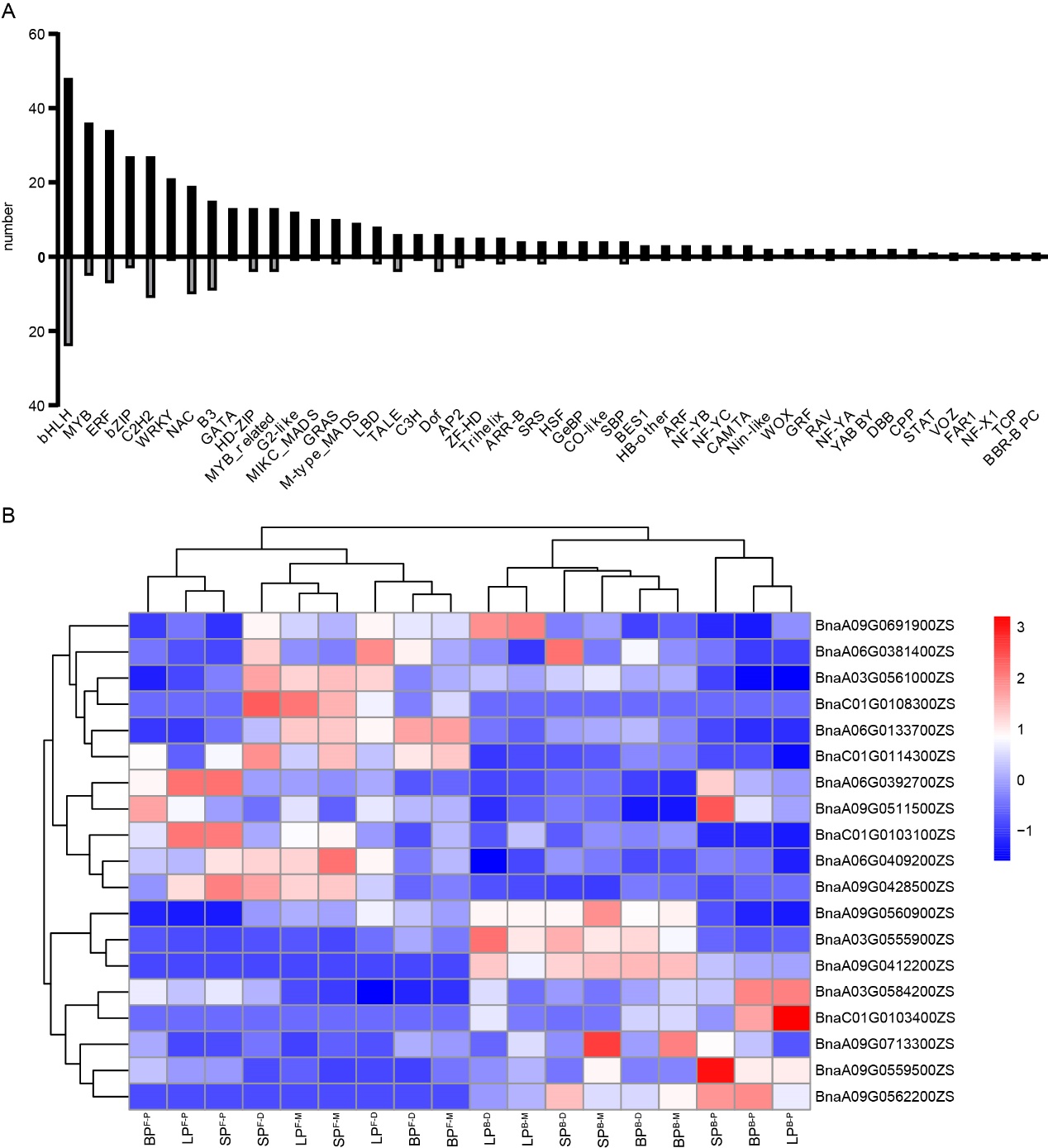


**Fig. S17. A** The distribution of all TFs (above the x-axis) and TFs correlated with PDRs (under the x-axis) within the five QTL hotspots that belong to different TF families. **B** The heat map of the expression of 19 high-confidential candidate TF genes that were expressed in petals, which was drawn using their FPKMs that normalized by z-score.
